# GRHL3 activates *FSCN1* to relax cell-cell adhesions between migrating keratinocytes during wound reepithelialization

**DOI:** 10.1172/jci.insight.142577

**Published:** 2021-09-08

**Authors:** Ghaidaa Kashgari, Sanan Venkatesh, Samuel Refuerzo, Brandon Pham, Anita Bayat, Rachel Herndon Klein, Raul Ramos, Albert Paul Ta, Maksim V. Plikus, Ping H. Wang, Bogi Andersen

**Affiliations:** 1 Department of Biological Chemistry, School of Medicine,; 2 Department of Developmental & Cell Biology, School of Biological Sciences, and; 3 Department of Medicine, Division of Endocrinology, School of Medicine, University of California, Irvine (UCI), California, USA.

**Keywords:** Dermatology, Genetics, Cell migration/adhesion, Skin

## Abstract

The migrating keratinocyte wound front is required for skin wound closure. Despite significant advances in wound healing research, we do not fully understand the molecular mechanisms that orchestrate collective keratinocyte migration. Here, we show that, in the wound front, the epidermal transcription factor Grainyhead like-3 (GRHL3) mediates decreased expression of the adherens junction protein E-cadherin; this results in relaxed adhesions between suprabasal keratinocytes, thus promoting collective cell migration and wound closure. Wound fronts from mice lacking GRHL3 in epithelial cells (*Grhl3*-cKO) have lower expression of Fascin-1 (FSCN1), a known negative regulator of E-cadherin. Assay for Transposase-Accessible Chromatin using sequencing (ATAC-seq) on wounded keratinocytes shows decreased wound-induced chromatin accessibility near the *Fscn1* gene in *Grhl3*-cKO mice, a region enriched for GRHL3 motifs. These data reveal a wound-induced GRHL3/FSCN1/E-cadherin pathway that regulates keratinocyte-keratinocyte adhesion during wound-front migration; this pathway is activated in acute human wounds and is altered in diabetic wounds in mice, suggesting translational relevance.

## Graphical abstract

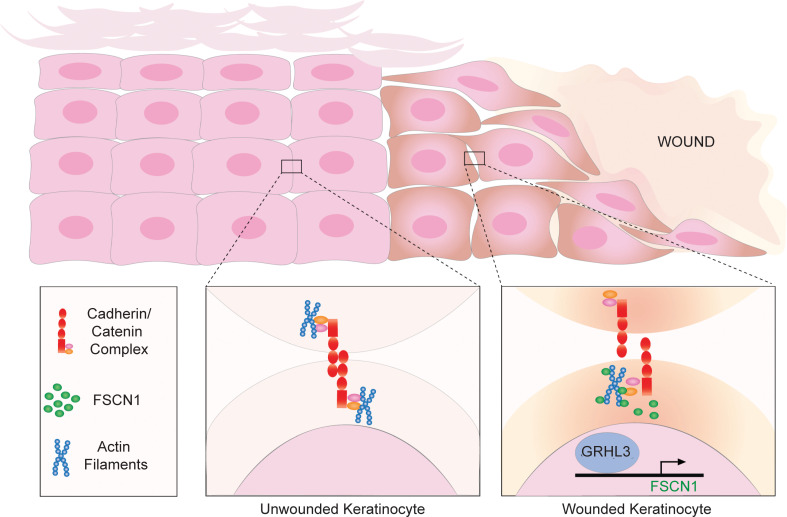

## Introduction

Acute skin wound healing progresses through 4 overlapping phases: hemostasis, inflammation, proliferation, and tissue remodeling (1). Although wounds close partially by dermal contraction, reepithelialization by epidermal keratinocytes in the proliferation phase is a key step in wound healing. During reepithelialization, keratinocytes in the wound front migrate on top of the underlying granulation tissue, ultimately meeting migrating keratinocytes from the opposing margin to close the defect (2). Migrating keratinocytes in the wound front are heterogenous and contain both basal and suprabasal subpopulations (3). Suprabasal keratinocytes in the intact epidermis express differentiation-specific keratins KRT1 and KRT10, whereas suprabasal keratinocytes in the wound front lose KRT1 and KRT10 expression and, instead, transiently express wound-specific keratins KRT6, KRT16, and KRT17 (3, 4).

Wound-front keratinocyte migration is an example of collective cell migration during which a group of cells moves as a cohesive unit. Changes in the actin-cytoskeleton and other traction forces, as well as the formation of cytoplasmic projections, contribute to active migration of leader cells at the wound edge (5, 6). These cellular changes are distinct from those occurring in the trailing suprabasal follower cells, which require loosening of cell-cell adhesions for effective migration (7). We have an incomplete understanding of the transcriptional mechanisms that mediate loosening of cell-cell adhesion in follower cells.

Grainyhead like-3 (GRHL3) is an evolutionarily conserved transcription factor required for mammalian development (8–10). Mice lacking *Grhl3* exhibit several abnormalities, including spina bifida, defective epidermal barrier, defective eyelid closure, and soft-tissue syndactyly (11–13). In addition to its role in epidermal differentiation, GRHL3 is required for periderm differentiation, preventing abnormal cell adhesion between adjacent epithelia (12). Although largely dispensable for adult skin homeostasis, GRHL3 is necessary for barrier recovery after epidermal injury (14, 15). In embryonic hind limb amputations, GRHL3 is highly expressed in cells in the wound margin, promoting actin-cytoskeleton contraction and wound closure (16). The work on GRHL3 in eyelid closure, embryonic wound healing, and in vitro keratinocyte migration indicates that it has roles in leader cells, but GRHL3’s potential role in follower cells has not been investigated. In addition, whereas GRHL3 is critical for the epidermal differentiation and barrier repair at the end stages of wound healing, its role in earlier stages of adult full-thickness wound healing has not been investigated.

FASCIN-1 (FSCN1) is an actin cross-linker that plays a critical role in cell migration (17, 18), promoting the formation of actin-based cellular protrusions required for cell motility, including filipodia. FSCN1 also regulates cell-cell adhesions in cancer cells, where it directly binds to microtubules (MT), regulating MT dynamics and adhesion stability during cell migration (19). Knockdown of *FSCN1* or blocking FSCN1-MT binding causes more stable adhesions and slower cell migration. By contrast, *FSCN1* overexpression in cancer cells is associated with E-cadherin downregulation and promotion of epithelial-mesenchymal transition (EMT) (20). Although it is known that *Fscn1* is upregulated in keratinocytes in the wound front (21, 22), its role in keratinocyte migration during wound healing has not been characterized.

Here, we show that GRHL3 is upregulated in the collectively migrating wound-front epidermis, most highly in the suprabasal compartment inhabited by follower keratinocytes. Loss of *Grhl3* leads to persistent cell-cell adhesions, abnormal architecture of the wound front, and delayed reepithelialization. We also identify as an underlying mechanism a new pathway where GRHL3 activates the *Fscn1* gene to effect cell-cell loosening in the migrating wound front, a pathway that is altered in diabetic wounds in mice.

## Results

### GRHL3 is upregulated in wound-front keratinocytes of acute wounds, and its expression is altered in human chronic wounds.

**Figure 1:**
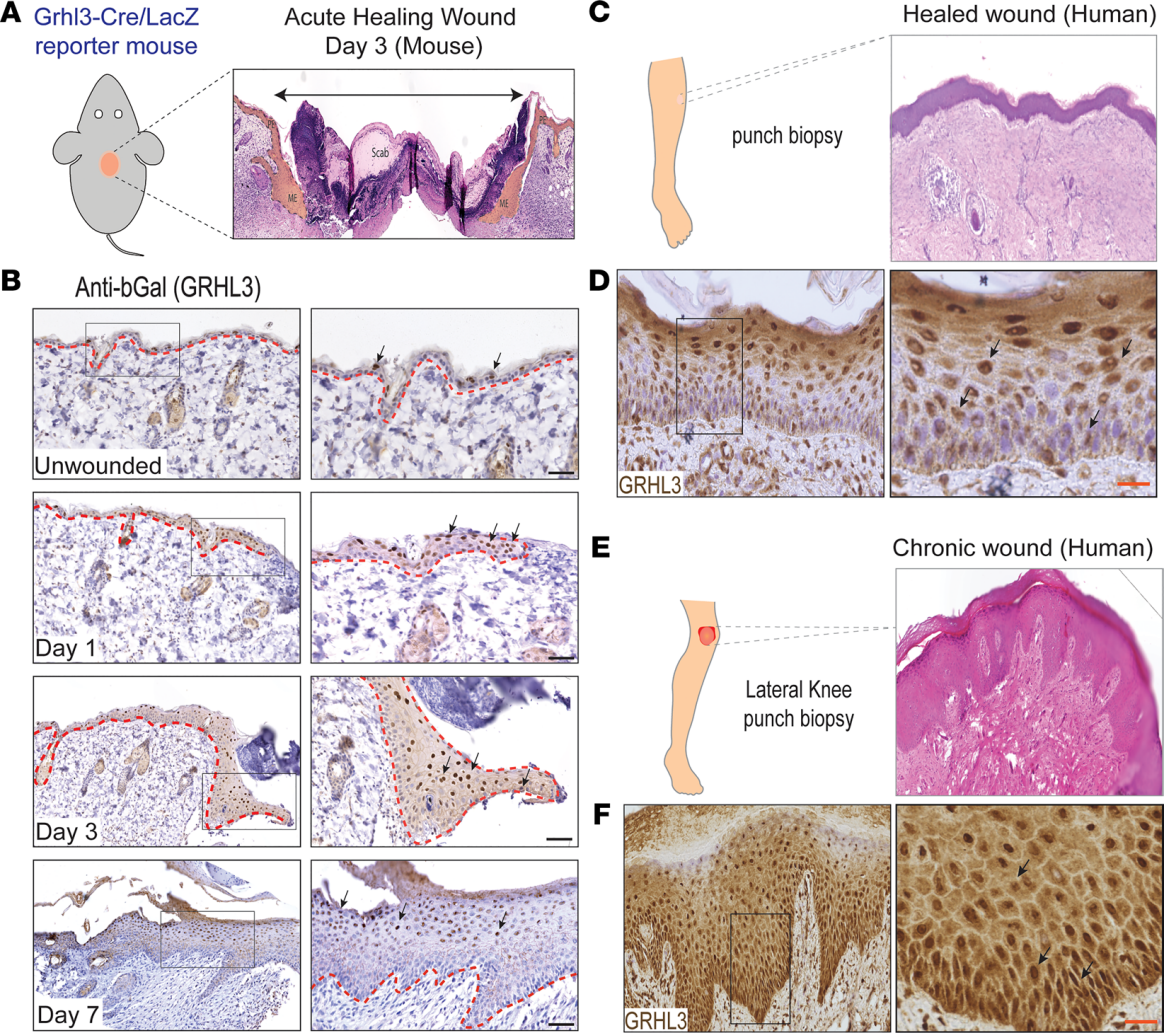
GRHL3 is upregulated in wound-front keratinocytes of acute wounds, and its expression is altered in human chronic wounds. (**A**) Schematic representation of the full-thickness mouse wound model for expression studies, showing mice reporting *Grhl3* expression with βGal (LacZ) expression (left panel). A wound showing location of the proliferating epidermis (PE), the migrating wound-front epidermis (ME), and a scab overlying the wound (right panel). (**B**) IHC localization of GRHL3 expression (anti-βGal antibody) in mouse skin sections at 0, 1, 3, and 7 days after wounding. Right panels show higher magnifications of the indicated areas in the black boxes. Scale bar: 60 μm. Black arrows point to the nuclear expression of GRHL3 in keratinocytes. (**C**) Schematic representation of a biopsy from a healing human wound (left panel). H&E staining of a healing human wound (right panel). (**D**) IHC staining for GRHL3 (anti-GRHL3 antibody) in a healing human wound. Right panel shows higher magnification of the indicated area in the black box. Black arrows point to the nuclear expression of GRHL3 in suprabasal cells in reepithelized human epidermis. Scale bar: 36 μm. (**E**) Schematic representation of a biopsy collected from a human chronic nonhealing wound (left panel). H&E staining of a human chronic nonhealing wound (right panel). (**F**) IHC staining for GRHL3 (anti-GRHL3 antibody) in a human chronic nonhealing wound. Right panel shows higher magnification of the indicated area in the black box. Black arrows point to the increased expression of GRHL3 in the deeper part of the chronic wound epidermis. Scale bar: 36 μm.

In mouse embryonic wounds, GRHL3 is upregulated at the wound leading edge where it regulates actin polymerization and cell polarity, mechanisms required for the purse-string closure of embryonic skin wounds (16). In adult skin, however, distinct mechanisms confer wound closure, including dermal contraction and collective migration of wound-front keratinocytes. Thus, we initiated our studies by defining the spatiotemporal expression of GRHL3 in full-thickness wounds in adult skin. We created small wounds (4 mm) on the back skin of *Grhl3*-Cre;LacZ reporter mice that have previously been shown to faithfully mark the endogenous expression of *Grhl3* in skin and other tissues (14). We preformed IHC analysis for β-Galactosidase (βGal) at several time points after wounding (Figure 1A; *n* = 3). In unwounded skin, βGal expression is sparse and within the suprabasal layer of the epidermis (Figure 1B). Twenty-four hours after wounding, when a migrating epithelium is forming, βGal is upregulated and more uniformly expressed in keratinocytes located in the wound front — in particular, suprabasally (Figure 1B). At day 3 after wounding, when keratinocyte migration is prominent, βGal is highly upregulated in migrating suprabasal keratinocytes in the wound front (Figure 1B). At day 7 after wounding, when epidermal cells have completed migration across the wound, increased expression of βGal persists in suprabasal keratinocytes (Figure 1B), consistent with the known role of GRHL3 in epidermal barrier restoration after injury (14). These data, showing upregulation of *Grhl3* in wound-front keratinocytes, are consistent with an active role of GRHL3 in keratinocyte migration during healing of acute wounds.

IHC analysis of GRHL3 in punch biopsies collected from healthy individuals with healing acute wounds also shows upregulation of GRHL3 in the wounded epidermis (Figure 1, C and D, and Supplemental Figure 1A; supplemental material available online with this article; https://doi.org/10.1172/jci.insight.142577DS1; *n* = 3). In contrast to mouse skin, in humans, we detected some GRHL3 expression in the basal layer, but as in the mouse, its highest expression is in the suprabasal layers of the epidermis, the regenerating spinous and granular layers (Figure 1D). Contrasting to acute healing wounds, chronic wounds (Figure 1, E and F, and Supplemental Figure 1A) have a higher proportion of basally located cells expressing GRHL3 (Supplemental Figure 1B; *n* = 3), although the total proportion of keratinocytes expressing GRHL3 is similar between healing and chronic wounds (Supplemental Figure 1C). Notably, basal keratinocytes in chronic wounds with high GRHL3 expression exhibit increased intercellular spaces and elongated, migratory-like cell morphology — features that are not observed in basal keratinocytes in acutely healing wounds (Figure 1F and Supplemental Figure 1A). Collectively, these data show that GRHL3 is upregulated in the wound front of healing wounds, consistent with a role in skin wound healing, and that GRHL3’s expression is abnormally increased in the basal layer in chronic human wounds.

### GRHL3 is required for the proper architecture and function of the collectively migrating wound front.

**Figure 2:**
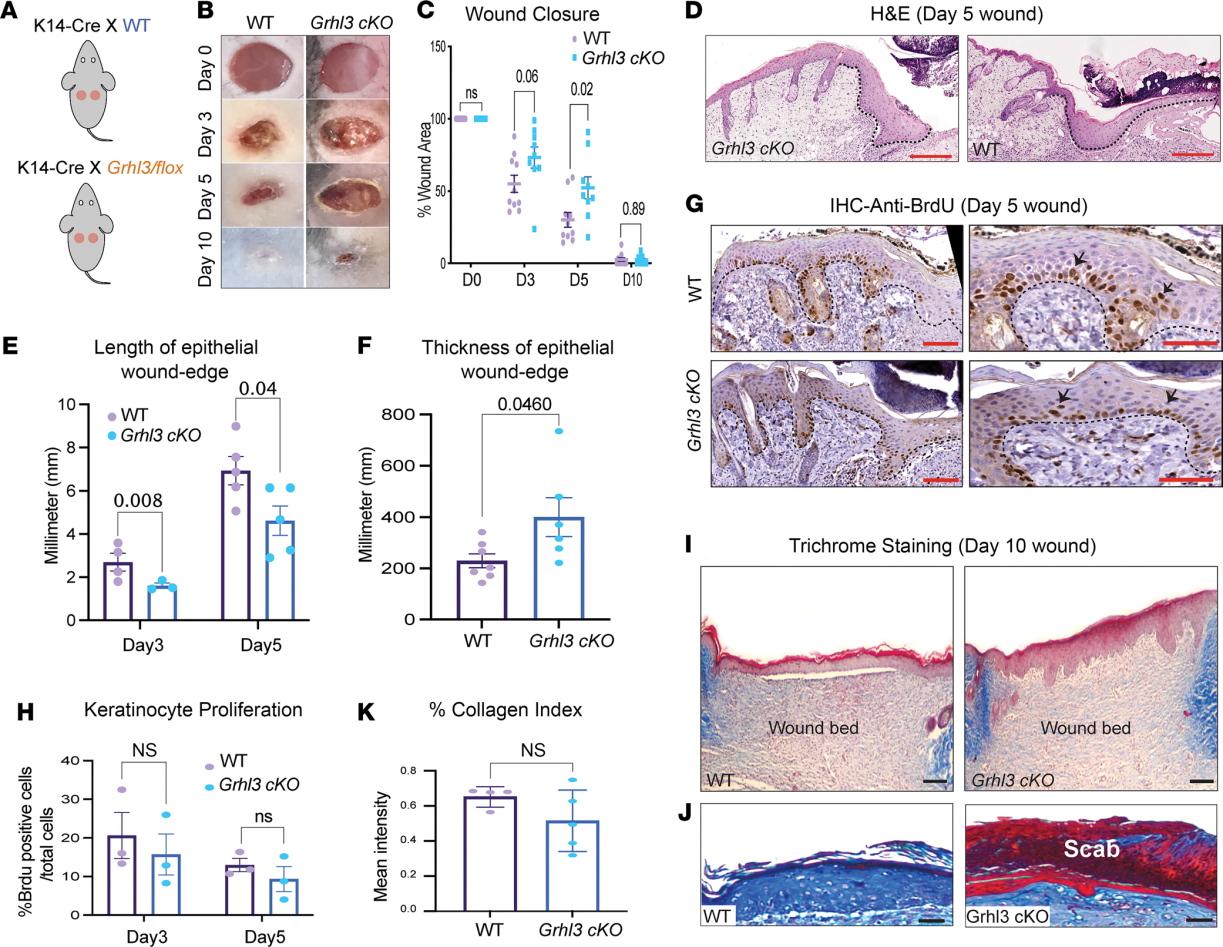
GRHL3 is required for proper architecture and function of the collectively migrating wound front. (**A**) Schematic representation of the full-thickness mouse wound model for healing studies. (**B**) Macroscopic images of full-thickness wounds at the indicated times after wounding in WT and *Grhl3*-cKO mice. (**C**) Wound size over time in WT and *Grhl3*-cKO mice (*n* = 5/genotype). (**D**) H&E staining of wound sections 5 days after wounding in WT and *Grhl3*-cKO mice. Dashed lines indicate the basal wound epithelium. Scale bar: 65 μm. (**E**) The length of the migrating epithelial wound front 3 and 5 days after wounding in WT and *Grhl3*-cKO mice (*n* = 3–5/genotype). (**F**) The thickness of the migrating epithelial wound front 5 days after wounding in WT and *Grhl3*-cKO mice (*n* = 3–5/genotype). (**G**) IHC staining with anti-BrdU antibody in wound sections 5 days after wounding in WT and *Grhl3*-cKO mice. Right panels show higher magnifications of the wounded epidermis. Dashed lines indicate the basal epithelium. Scale bar: 33 μm. Black arrows indicate BrdU^+^ cells. (**H**) Percentage of BrdU^+^ cells in the wound-front epidermis at days 3 and 5 after wounding in WT and *Grhl3*-cKO mice (*n* = 3–5/genotype). (**I**) Masson’s trichrome staining of wound sections 10 days after wounding in WT and *Grhl3*-cKO mice. Scale bar: 80 μm. (**K**) Percentage of measured collagen index in the wound bed 10 days after wounding in WT and *Grhl3*-cKO mice (*n* = 3/genotype). (**J**) Higher magnification images of Masson’s trichrome staining in day 10 wound sections. Note the attachment of a scab to the wounded epidermis in *Grhl3*-cKO mice. Scale bar: 40 μm. Statistical significance was determined using 1-way ANOVA (**C**, **E**, and **H**) and Student’s *t* test (**F** and **K**). Data are presented as mean ± SEM.

To investigate the role of GRHL3 in adult skin wound healing, we studied 4 mm punch wounds on the back of WT mice and mice conditionally deleted for *Ghrl3* in keratinocytes (*Grhl3*-cKO) (Figure 2A; *n* = 5/genotype). The deletion efficiency of *Grhl3* in these mice was validated with quantitative PCR (qPCR) primers amplifying the region between the *Grhl3* loxP sites (Supplemental Figure 2A) and with GRHL3 immunostaining of the wound front in *Grhl3*-cKO mice (Supplemental Figure 2B). The size of the wounds was examined at several time points: 0, 3, 5, and 10 days after wounding. Macroscopic analysis of the wounds over time showed delayed wound closure in the *Grhl3*-cKO mice, as indicated by a significantly increased wound size compared with WT mice at day 3 and day 5 (Figure 2, B and C). Histological analysis of wound sections collected at the same time points showed significantly decreased length of the migrating epithelial wound front in *Grhl3*-cKO mice compared with WT mice (Figure 2, D and E). In addition, the height (thickness) of the migrating epithelial wound front was significantly increased in *Grhl3*-cKO mice compared with WT mice at day 5 (Figure 2F). The delayed wound closure and the altered architecture of the migrating epithelial wound front in *Grhl3*-cKO mice align with the spatiotemporal upregulation of GRHL3 in wound-front keratinocytes during the reepithelialization phase of wound healing. Together, these data indicate that GRHL3 is required for proper wound reepithelialization.

Wound reepithelialization involves 2 independent processes: proliferation of keratinocytes located distally from the wound front and collective migration of keratinocytes located in the wound front. Thus, we determined whether decreased keratinocyte proliferation could explain the delayed progression of the wound front in the *Grhl3*-cKO mice. BrdU staining of wound sections collected at day 3 and day 5 after wounding indicated no significant differences in the number of proliferative cells between *Grhl3*-cKO and WT mice (Figure 2, G and H; *n* = 3–4/genotype), suggesting that altered keratinocyte migration rather than decreased cell proliferation causes delayed wound closure in the *Grhl3*-cKO mice.

Wounds in both WT and *Grhl3*-cKO mice were closed by day 10 (Figure 2, B and C), suggesting that lack of epidermal GRHL3 does not affect dermal contraction. Indeed, Masson’s trichrome staining showed no significant differences between the 2 genotypes in collagen fiber deposition in the wound bed at day 10 (Figure 2, I–K; *n* = 3–4/genotype). By contrast, there was a clear increase in the thickness of the epidermis covering the *Grhl3*-cKO wounds at day 10 (Figure 2I). Also, after complete wound closure at day 10, wound scab remained attached to the epidermis in *Grhl3*-cKO mice, a phenomenon associated with poor reepithelialization (Figure 2J). Together, these data suggest that defective collective keratinocyte migration impairs wound closure in mice deleted for *Grhl3* in the epidermis.

### GRHL3 mediates decreased E-cadherin expression and loosening of cell-cell adhesions in the migrating wound front.

**Figure 3:**
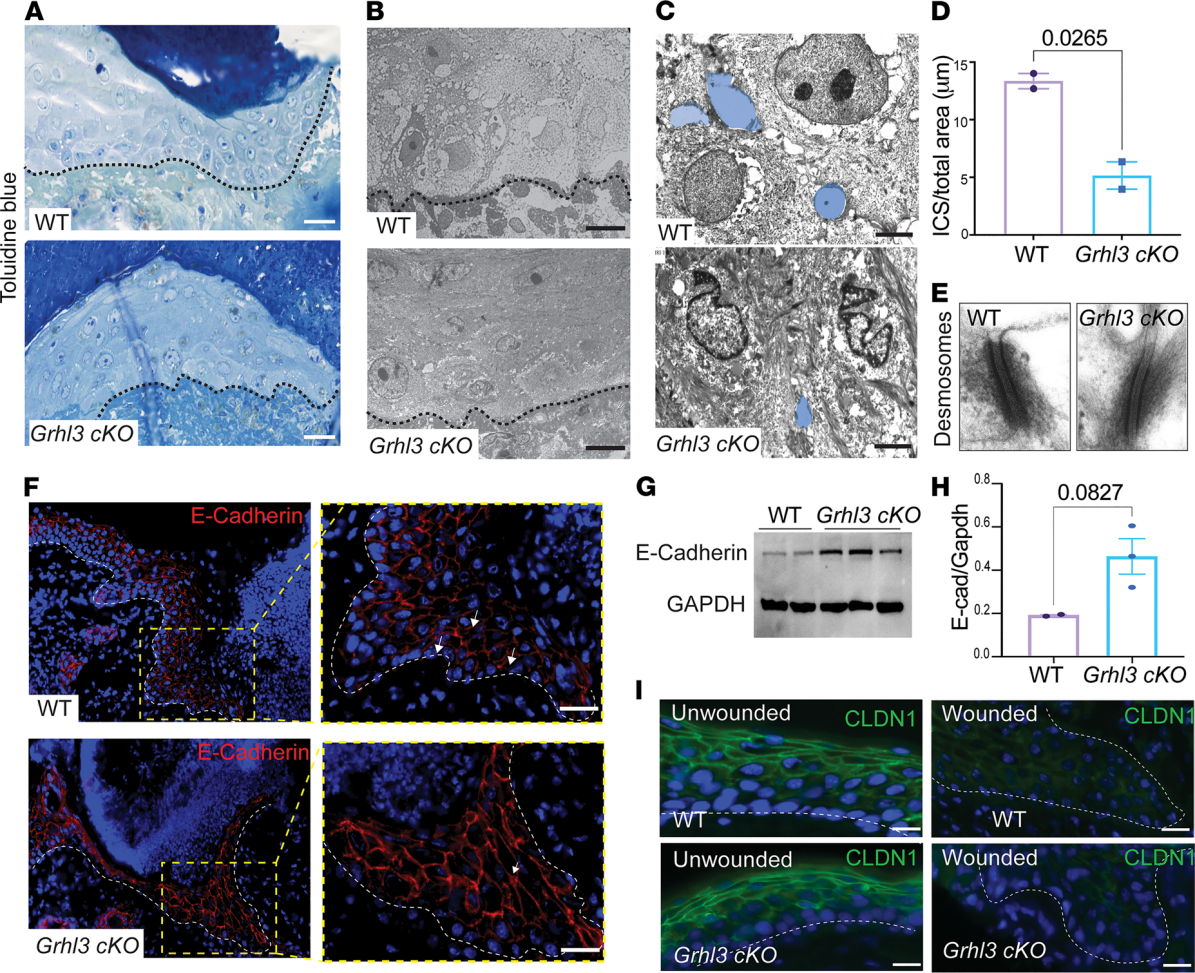
GRHL3 mediates decreased E-cadherin expression and loosening of cell-cell adhesion in the migrating wound front. (**A**) Toluidine blue staining of resin embedded semi-thin (100 nm) wound sections at day 3 after wounding. Dotted lines indicate the basis of the migrating wound epithelium. Scale bar: 36 μm. (**B**) Transmission electron microscopy (TEM) of the wound front at day 3 in WT and *Grhl3*-cKO mice. Dotted lines indicate the basement membrane. Scale bar: 10 μm. (**C**) Higher-magnification TEM images showing individual cells at the wound front in WT and *Grhl3*-cKO mice 3 days after wounding. Scale bar: 2 μm. Light blue filling indicates the intercellular spaces between 2 migrating keratinocytes at the wound front. (**D**) The area covered by intercellular spaces (data are presented as mean ± SD) between migrating keratinocytes at the wound front in **C** (*n* = 2/genotype). (**E**) Higher-magnification TEM images showing desmosomal junctions between migrating keratinocytes at the wound front in WT and *Grhl3*-cKO mice. Scale bar: 100 nm. (**F**) Immunofluorescence analysis of E-cadherin (anti–E-cadherin) in day 3 wound sections collected from WT and *Grhl3*-cKO mice. Dotted lines indicate the basis of the wound front epithelium. Right panels show higher magnification of migrating keratinocytes at the wound front. Arrows show cell surface expression of E-cadherin in WT mice and increased accumulation of E-cadherin in *Grhl3*-cKO mice. Scale bar: 30 μm. (**G**) Western blot analysis of E-cadherin protein and loading control (GAPDH) in lysates isolated from wound margins at day 3 in WT and *Grhl3*-cKO mice (*n* = 2–3/genotype). (**H**) Quantifications of Western blot band intensity (data are the mean ± SEM) of E-cadherin and GAPDH proteins in **G**. (**I**) Immunofluorescence analysis of Claudin-1 (anti-CLDN1) expression in day 3 wound sections collected from WT and *Grhl3*-cKO mice. Scale bar: 33 μm. Dotted lines indicate the basis of the wound epithelium (left) and the basement membrane of the unwounded epidermis (right). Statistical significance was determined using Student’s *t* test (*P < 0.05).

We next wanted to further characterize the defect in the collectively migrating epithelial wound front in *Grhl3*-cKO mice. Toluidine blue staining of thin wound sections (100 nm) showed clear intercellular spaces (faintly stained white areas) between migrating keratinocytes at the wound front in WT mice at day 3 after wounding (Figure 3A, top panel). By contrast, in the *Grhl3*-cKO wound front, we barely detected intercellular gaps between migrating keratinocytes, especially between suprabasal keratinocytes (Figure 3A, bottom panel). The closely packed keratinocytes in the *Grhl3*-cKO wound front also exhibit a larger cytoplasmic area than keratinocytes in the WT wound front (Figure 3A). Furthermore, we observed significantly decreased intercellular spaces in *Grhl3*-cKO mice, using higher-resolution transmission electron microscopy (TEM) images of the collectively migrating wound front at day 3 (Figure 3, B–D; *n* = 2/genotype). These observations suggest that increased keratinocyte-keratinocyte adhesions in the collectively migrating wound front cause its abnormal architecture and delayed progression during wound healing in *Grhl3*-cKO mice.

In the collectively migrating wound front, components of adherens and tight junctions, but not desmosomal junctions, are downregulated (7). This downregulation causes loosening of adhesions between migrating cells, which is required for collective cell migration (7, 23). Consistent with these observations by others, we found no changes in keratinocyte desmosomal junctions in the wound front in *Grhl3*-cKO mice (Figure 3E). By contrast, and in line with previous findings (23), the adherens junction component E-cadherin was downregulated in basal keratinocytes in the collectively migrating wound front in both WT and *Grhl3*-cKO mice (Figure 3F). However, compared with WT mice, cell-surface levels of E-cadherin were increased in suprabasal keratinocytes of the *Grhl3*-cKO wound front (Figure 3F and Supplemental Figure 3, A and B; *n* = 3/genotype). These observations were further supported by Western blot analysis showing a significant increase of E-cadherin protein levels in *Grhl3*-cKO wound lysates compared with WT at 3 days after wounding (Figure 3, G and H), suggesting altered adherens junctions in *Grhl3*-cKO wounds. By contrast, the expression of the tight-junction component Claudin-1 (Cldn1) is similarly downregulated in keratinocytes in the wound fronts of *Grhl3*-cKO and WT mice (Figure 3I). Together, these data indicate that, in response to wounding, GRHL3 mediates decreased E-cadherin expression and loosening of adherens junctions required for normal architecture and movement of the migrating epithelial wound front.

### GRHL3 regulates the expression of factors that modulate E-cadherin levels and cell-cell adhesions in the wound front.

**Figure 4:**
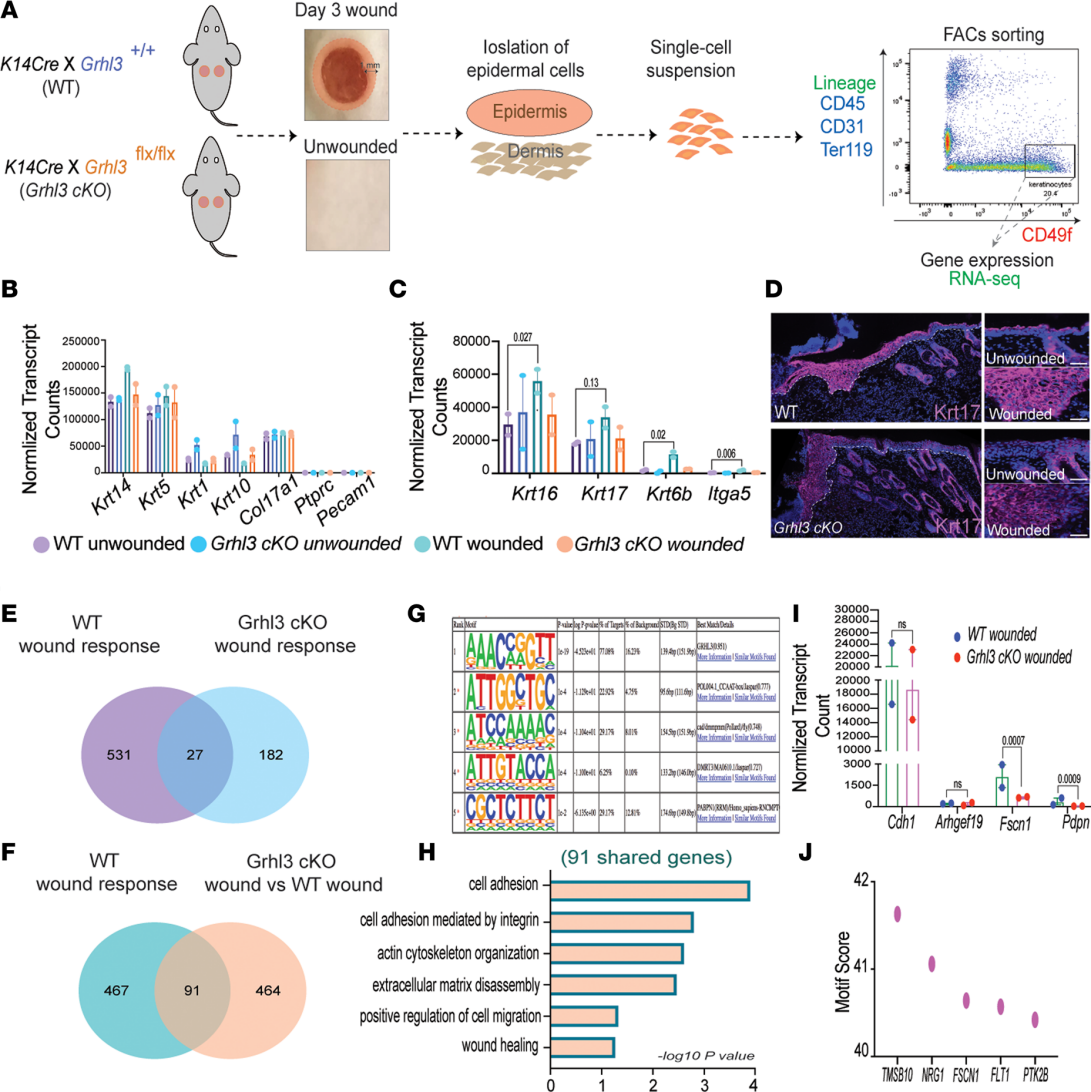
GRHL3 regulates the expression of factors that modulate E-cadherin levels and cell-cell adhesion in the wound front. (**A**) Schematic representation of wound-front keratinocyte isolation at day 3 from WT and *Grhl3*-cKO mice (*n* = 2/genotype). (**B**) Normalized mRNA expression (reads per kilobase of transcript, per million mapped reads [RPKM]) of keratinocyte and nonkeratinocyte markers (mean ± SEM). (**C**) Normalized mRNA expression (RPKM) of wound markers (mean ± SEM). (**D**) Immunofluorescence analysis of Keratin-17 (anti-Krt17) expression in wound sections from WT and *Grhl3*-cKO mice. Dotted lines indicate the epidermis-dermis junctions. Side panels show higher magnification of Krt17 expression in unwounded and wounded epidermis. Scale bar: 45 μm. (**E**) Venn diagrams depicting the overlap of genes that are differentially expressed in the WT wound response (WT wounded versus unwounded) and the genes that are differentially expressed in the *Grhl3*-cKO wounds response (*Grhl3*-cKO** wounded versus unwounded). (**F**) Venn diagrams depicting the overlap of genes that are differentially expressed in WT wound response (WT wounded versus unwounded) and the genes that are differentially expressed in *Grhl3*-cKO wounds (*Grhl3*-cKO** wounded versus WT wounded). (**G**) Motif analysis on overlapped genes (91 genes from **F**) that are differentially expressed in the WT wound response and *Grhl3*-cKO wounds. (**H**) Gene ontology of differentially expressed genes (91 overlapped genes from **F**) in the WT wound response and *Grhl3*-cKO wounds. (**I**) Normalized mRNA expression (RPKM; mean ± SEM) of genes associated with cell adhesion and E-cadherin regulation in wounded keratinocytes in WT and *Grhl3*-cKO mice 3 days after wounding (*n* = 2/genotype). (**J**) Selected differentially expressed genes in *Grhl3*-cKO wounded keratinocytes with high GRHL3 motif score. Statistical significance was determined using Kallisto (*P < 0.05).

Our findings suggest that GRHL3, directly or indirectly, downregulates the expression of E-cadherin in migrating keratinocytes at the wound front during reepithelialization. To understand the underlying mechanisms, we performed transcriptomic analysis on isolated wound-front keratinocytes from WT and *Grhl3*-cKO mice; we collected 1 mm of the wound margin at day 3 and separated the epidermis from the dermis using enzymatic digestion (Figure 4A; *n* = 2/genotype). We employed FACS to isolate keratinocytes, using the common keratinocyte surface marker integrin α-6 (CD49F); nonkeratinocytes — immune cells, endothelial cells, and hematopoietic cells — were excluded using lineage surface markers. To capture both basal and suprabasal keratinocytes, we sorted keratinocytes that express CD49F at intermediate (1 × 10^3^ to 1 × 10^4^) and high (> 1 × 10^5^) levels (Supplemental Figure 4A). Keratinocytes from unwounded regions of the skin were collected as a control (Figure 4A).

The FACS strategy was successful, as normalized mRNA expression showed enrichment of keratinocyte markers *Krt14*, *Krt5*, *Krt1*, *Krt10*,** and *Col17a1*, with no expression of nonkeratinocyte markers in wounded and unwounded samples of both genotypes (Figure 4B). The expression of wound-response markers *Krt16*, *Krt17*, and *Krt6b*, and the leading-edge marker *Itga5*, was markedly upregulated in keratinocytes isolated from the wound front compared with keratinocytes isolated from unwounded epidermis in WT mice (Figure 4C), validating the isolation of 2 distinct keratinocyte populations: unwounded and wounded. By contrast, the expression of wound-response markers was unchanged between wounded and unwounded keratinocytes from *Grhl3*-cKO mice (Figure 4C), suggesting a general defect in the wound response in the absence of *Grhl3*. In agreement with these findings, immunostaining of day 3 wounds showed markedly less robust upregulation of KRT17 expression in keratinocytes at the wound front in *Grhl3*-cKO mice compared with that in the WT (Figure 4D and Supplemental Figure 4B; *n* = 2/genotype); note that the expression of KRT17 in other compartments such as hair follicles is comparable between the 2 genotypes. These data suggest that GRHL3 is required for activation of major components of the wound response in wound-front keratinocytes.

In the WT wound response, there were 558 genes differentially expressed (*P* < 0.05) between wound-front keratinocytes and unwounded keratinocytes (Figure 4E and Supplemental Table 1). By comparison, in the *Grhl3*-cKO wound response, there were 209 genes differentially expressed (*P* < 0.05) between wound-front keratinocytes and unwounded keratinocytes (Figure 4E and Supplemental Table 2). Comparing wound response gene expression profiles between WT and *Grhl3*-cKO, only 27 genes were shared between the 2 genotypes, 531 genes were exclusive to the WT wound response, and 182 genes were exclusive to the *Grhl3*-cKO wound response (Figure 4E and Supplemental Table 3). GO categories of wound response genes that are differentially expressed only in WT mice are associated with cell adhesion, angiogenesis, hypoxia, and wounding (Supplemental Figure 4C). In contrast, GO categories of wound response genes that are differentially expressed only in *Grhl3*-cKO mice are associated with angiogenesis, keratinocyte differentiation, and response to progesterone (Supplemental Figure 4D). Additionally, shared wound response genes between the 2 genotypes showed enrichment of genes associated with extracellular matrix disassembly, cell migration, and cell adhesion (Supplemental Figure 4E). Collectively, these data indicate that upregulation of GRHL3 in wound-front keratinocytes is important for the changes in expression of a large subset of normal wound-response genes.

When we compared gene expression profile of (*Grhl3*-cKO wounded versus WT wounded) keratinocytes with gene expression profile of (WT wounded versus WT unwounded) keratinocytes, there are only 91 genes that are affected in both genotypes in response to wounding (Figure 4F and Supplemental Table 4). Motif analysis on the shared genes (91 genes in Figure 4F) shows high enrichment of the GRHL3 motif, suggesting possible direct targets that are regulated by GRHL3 in response to wounding (Figure 4G). Gene ontology (GO) analysis of these shared genes (91 genes in Figure 4F) are associated with cell adhesion, cell migration, and wounding (Figure 4H). Interestingly, mRNA levels of the cell adhesion gene *Cdh1* (encoding E-cadherin) and cell migration gene *Arhgef19,* a previously described downstream target of Grhl3 (16), were unchanged in *Grhl3*-cKO mice compared with WT mice after wounding (Figure 4I and Supplemental Figure 4F). In contrast, mRNAs encoding 2 wound-induced factors that are known to negatively regulate E-cadherin expression — *Fscn1* (21) and Podoplanin (*Pdpn*) (24) — were significantly downregulated in *Grhl3*-cKO mice compared with WT mice after wounding (Figure 4I). These data indicate that GRHL3 does not transcriptionally stimulate the expression of *Cdh1* in wound-front keratinocytes and that its regulation of E-cadherin levels may be via other proteins. In fact, motif analysis of genes that are differentially expressed in response to wounding in the *Grhl3*-cKO mice show high enrichment of GRHL3 motifs, including in the *Fscn1* gene (Figure 4J), a candidate intermediary between GRHL3 and E-cadherin. These data indicate that, in wound-front keratinocytes, GRHL3 regulates the expression of genes encoding proteins that modulate E-cadherin levels and cell-cell adhesions during keratinocyte migration.

### GRHL3 upregulates FSCN1 in migrating wound-front keratinocytes.

**Figure 5:**
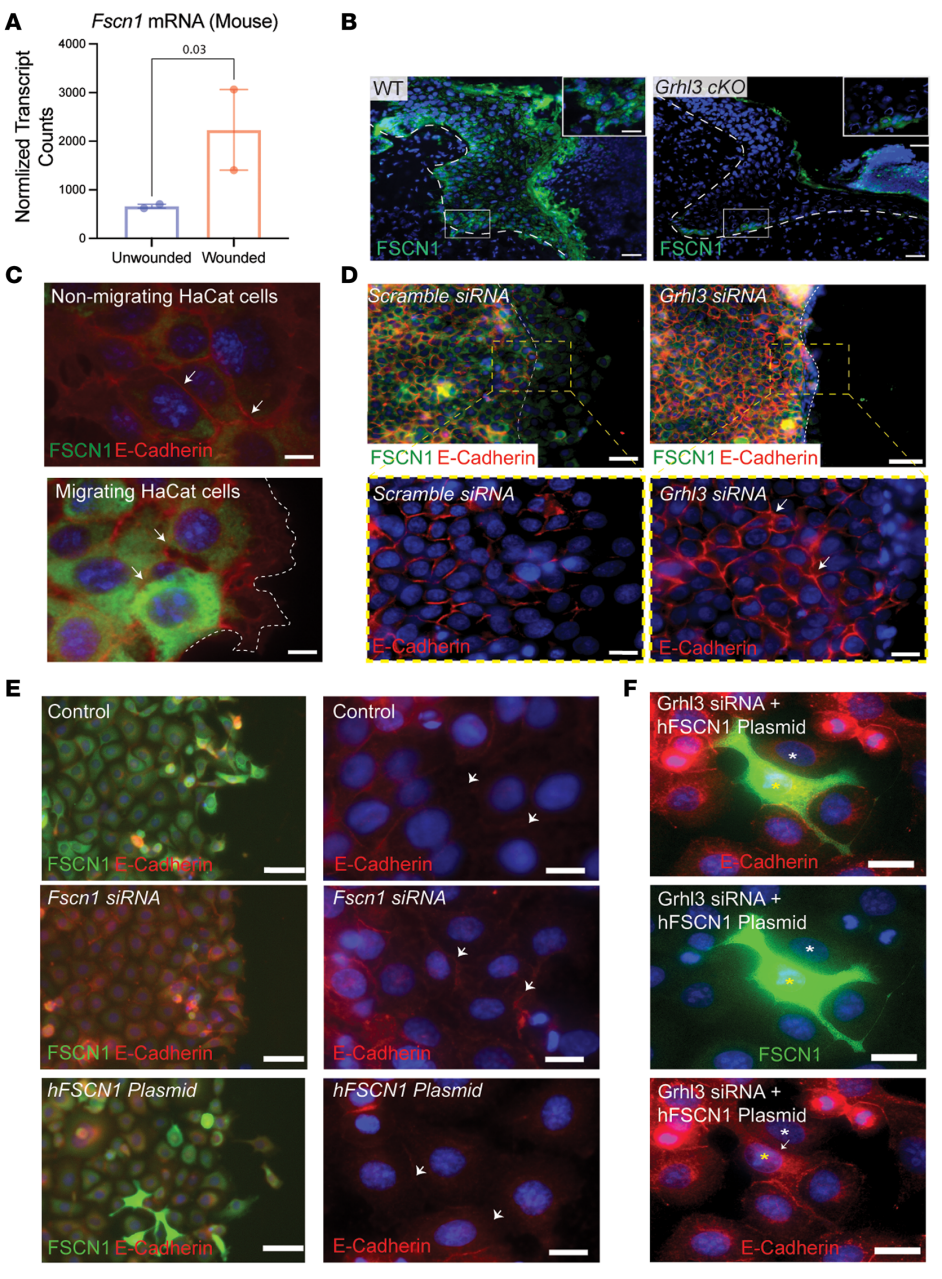
GRHL3 upregulates FSCN1 in migrating wound-front keratinocytes. (**A**) Normalized *Fscn1* mRNA expression (RPKM; mean ± SEM) in unwounded and wounded keratinocytes in WT mice 3 days after wounding (*P < 0.05). (**B**) Immunofluorescence analysis of FSCN1 (anti-FSCN1) expression in day 3 wound sections in WT and *Grhl3*-cKO mice. Dashed lines indicate the basis of the wound front. White boxes indicate the areas that are magnified on the top right. Scale bar: 40 μm. (**C**) Immunofluorescence analysis of FSCN1 (green), E-cadherin (red), and DAPI (blue) in nonmigrating and migrating HaCaT cells 12 hours after scratch. Scale bar: 10 μm. White arrows point to E-cadherin expression. Dashed lines indicate the leading front (right panel). (**D**) Immunofluorescence analysis of FSCN1 (green), E-cadherin (red), and DAPI (blue) in migrating HaCaT cells at the leading edge in cells transfected with scramble siRNAs (upper) and *Grhl3* siRNAs (lower). Dashed lines indicate the original scratch area. Scale bar: 150 μm. Higher magnification of the area in the demarcated boxes (left panels) showing immunofluorescence staining of E-cadherin (red) and DAPI (blue) in migrating HaCaT cells transfected with scramble siRNAs and *Grhl3* siRNAs (right panels). Scale bar: 30 μm. (**E**) Immunofluorescence staining of *FSCN1* (left column) and E-cadherin (right column) in migrating HaCaT cells transfected with scramble siRNAs (control), *FSCN1* siRNAs, and h*Fscn1(*pRP-EGFP-hFSCN1). Scale bars: 30 μm (left) and 10 μm (right). White arrows point to E-cadherin expression at the cell membrane. (**F**) Immunofluorescence analysis of FSCN1 (green), E-cadherin (red), and DAPI (blue) in HaCaT cells transfected with *GRHL3* siRNAs and *pRP-EGFP-hFSCN1* plasmid. The white asterisk indicates a HaCaT cell with high FSCN1 expression. The yellow asterisk indicates a HaCaT cell with low FSCN1 expression. The white arrow points to the absent E-cadherin protein at the cell membrane. The yellow arrow points to E-cadherin at the cell membrane. Scale bar: 10 μm.

In cancer cells, FSCN1 upregulation is associated with E-cadherin downregulation, decreased cell-cell adhesion, and increased migration (25), prompting us to investigate the role of GRHL3 in *Fscn1* regulation in wound-front keratinocytes during reepithelialization. In WT mice, wounding significantly induced *Fscn1* mRNA expression in keratinocytes (Figure 5A). Consistent with the mRNA findings, immunostaining of wound sections at day 3 after wounding showed high expression of FSCN1 in keratinocytes in the wound front, with highest expression in basal keratinocytes (Figure 5B) where E-cadherin expression is undetectable (Figure 3F). By contrast, in *Grhl3*-cKO mice, FSCN1 protein is detected in low levels in basal keratinocytes and is markedly downregulated in suprabasal keratinocytes in the wound front (Figure 5B and Supplemental Figure 5A), consistent with the significant downregulation in *Fscn1* mRNA expression in sorted wound-front keratinocytes (Figure 4I). Together, these data suggest that GRHL3 upregulates *Fscn1* in keratinocytes of the migrating wound front.

Next, we investigated the expression of FSCN1 in relation to E-cadherin during HaCaT keratinocyte migration in vitro. Twelve hours after a scratch in confluent cultures (Figure 5C and Supplemental Figure 5B), FSCN1 expression was upregulated in cells located at the migrating front with low expression in nonmigrating cells located away from the edge (Figure 5C). By comparison, E-cadherin expression in nonmigrating cells that have low FSCN1 expression remained intact, whereas in cells at the migrating front that have high FSCN1 expression, E-cadherin expression was patchy, consistent with the migratory cell morphology (Figure 5C, white arrows). These data demonstrate that FSCN1 upregulation correlates negatively with E-cadherin expression in keratinocytes.

We also investigated the effect of *GRHL3* knockdown (Supplemental Figure 5C) on FSCN1 and E-cadherin expression in the migrating human keratinocyte front in vitro. As expected (15, 16), *GRHL3* knockdown impaired HaCaT cell migration, but it also downregulated FSCN1 expression and increased E-cadherin protein in migrating HaCaT cells (Figure 5D and Supplemental Figure 5, D and E). Together, these findings suggest that GRHL3 relaxes cell-cell by upregulating the expression of *FSCN1*, a negative regulator of E-cadherin, in wound-front keratinocytes from mice and humans.

To test this hypothesis further, we investigated the expression of E-cadherin in relation to *FSCN1* downregulation and upregulation during keratinocyte migration in vitro (Figure 5E). In support of our hypothesis, *FSCN1* knockdown resulted in increased E-cadherin at the cell surface (Figure 5E, middle panel), whereas *FSCN1* overexpression decreased E-cadherin at the cell surface (Figure 5E, bottom panel). Furthermore, transfected HaCaT cells that express FSCN1 to a high level show absence of E-cadherin at the cell membrane, whereas neighboring HaCaT cells that express lower levels of FSCN1 maintained the expression of E-cadherin at the cell membrane (Supplemental Figure 5F). Lastly, we tested whether forced expression of *FSCN1* in HaCaT cells knocked down for *GRHL3* would counteract the increased E-cadherin expression after *GRHL3* knockdown (Figure 5F). In support of our model, we observed that cells expressing FSCN1 to a high level lack E-cadherin at their cell membrane, whereas adjacent *GRHL3* knockdown cells that express lower levels of FSCN1 maintain E-cadherin expression at the cell membrane (Figure 5F). Together, these data support a role for GRHL3/FSCN1/E-cadherin pathway activation in migrating keratinocytes at the wound front.

### The FSCN1/E-cadherin pathway is activated in human acute wounds and impaired in diabetic mouse wounds.

**Figure 6:**
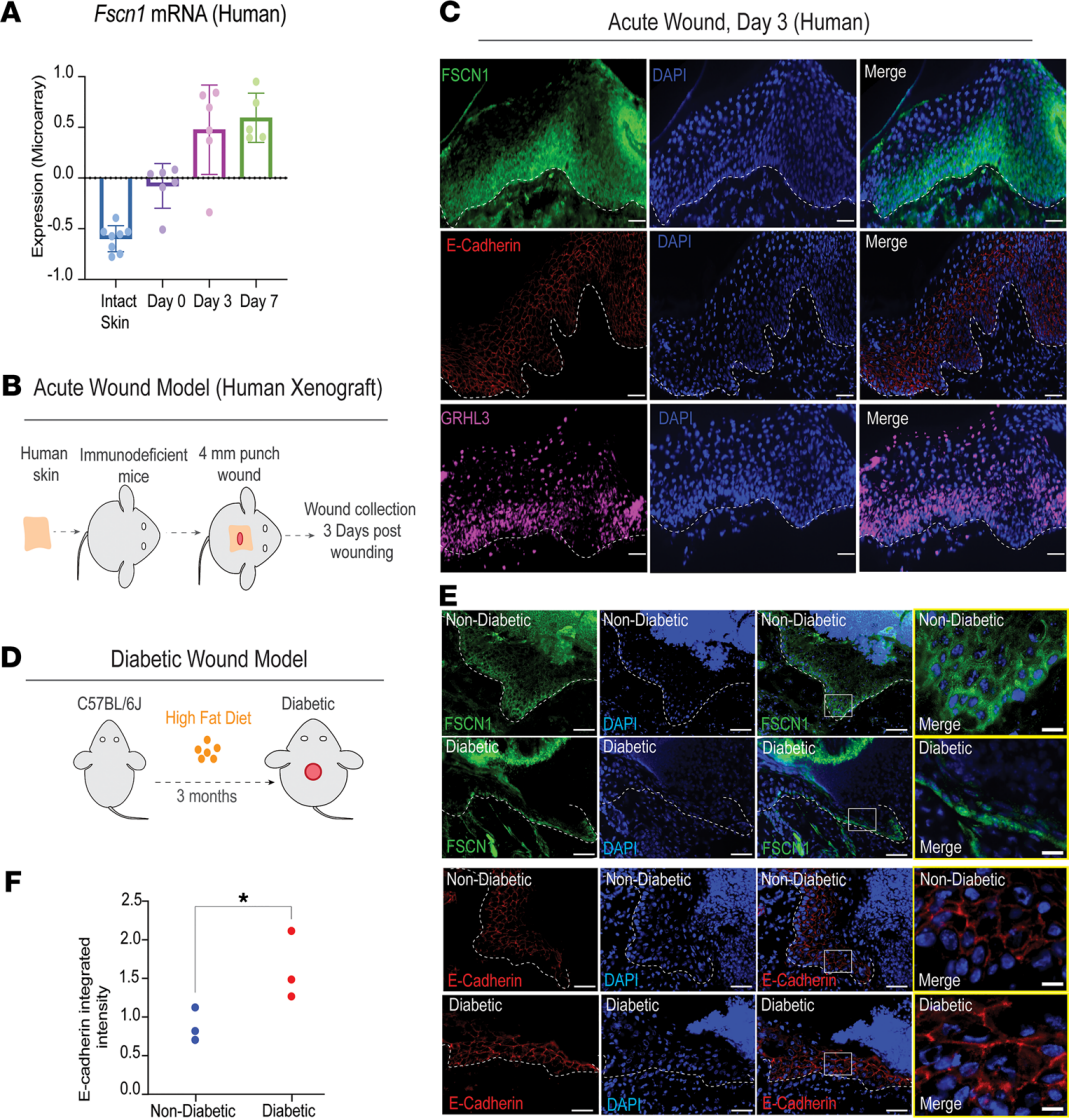
The GRHL3/FSCN1/E-cadherin pathway is activated in acute human wounds and impaired in diabetic mouse wounds. (**A**) *FSCN1* mRNA expression (mean ± SEM) in acute human wounds at the indicated times after wounding. (**B**) Schematic representation of the acute wound model using human skin xenografts (*n* = 3). (**C**) Immunofluorescence analysis of FSCN1 (green), E-cadherin (red), and GRHL3 (magenta) in human wounds at day 3 after wounding. Dashed lines indicate the basis of the migrating epithelium in the wound front. Scale bar: 70 μm. (**D**) Schematic representation of the mouse diabetic wound model (*n* = 3). (**E**) Immunofluorescence analysis of FSCN1 (green), E-cadherin (red), and DAPI (blue) in nondiabetic and diabetic mice at day 3 after wounding. Scale bar: 200 μm. Dashed lines indicate the basis of the migrating epithelium in the wound front. White boxes indicate the area of magnified images (rightmost panel). Scale bar: 45 μm. (**F**) Integrated intensity of E-cadherin staining in nondiabetic and diabetic wound sections (*n* = 3). Statistical significance was determined using Student’s t test (*P < 0.05).

Mining previously published gene expression data from human acute wounds at several time points (26), we found that *FSCN1* mRNA expression is upregulated 3 and 7 days after wounding, concomitant with wound reepithelialization (Figure 6A). To better characterize the expression of FSCN1 in relation to GRHL3 in human acute wounds, we xenografted surgically discarded human skin isolated from healthy subjects onto the dorsal back of immunodeficient mice (Figure 6B; *n* = 3). Following recovery, a small wound (2 mm) was created on the xenograft, and wound tissue was collected 3 days after wounding, during the period of active reepithelialization (Figure 6B). We then used immunostaining to study the expression of E-cadherin, FSCN1, and GRHL3 in these acute human wounds. Consistent with our mouse results, E-cadherin and FSCN1 expression was largely complementary in keratinocytes at the wound front; FSCN1 was upregulated in the wound front, with higher expression in basal keratinocytes where E-cadherin expression was almost absent (Figure 6C). GRHL3 was also upregulated in keratinocytes in the wound front, with positive correlation with FSCN1 expression (Figure 6C). Taken together with our mouse data, these experiments suggest that the GRHL3/FSCN1/E-cadherin pathway is activated during reepithelialization of acute wounds in both mice and humans.

Since GRHL3 is highly expressed in human chronic wounds (Figure 1), we next investigated whether components of the GRHL3/FSCN1/E-cadherin pathway are altered in mouse diabetic wounds, a model of delayed wound healing where mice are made diabetic through high-fat feeding (27) (Figure 6D; *n* = 3); diabetes was confirmed with fasting glucose measurements (fasting blood glucose > 200 mg/dL). Although FSCN1 was detected in some basal keratinocytes in diabetic wounds, it was downregulated in suprabasal keratinocytes in the wound front compared with controls (Figure 6E and Supplemental Figure 6A). Consistent with this finding, we observed more E-cadherin expression at the cell surface in keratinocytes at the diabetic wound front, similar to the E-cadherin expression pattern in *Grhl3*-cKO wounds (Figure 6, E and F). We did not observe alterations in GRHL3 protein expression in wound sections collected from diabetic mice compared with control mice (data not shown), suggesting that the changes in *Fscn1* expression may be independent of GRHL3 in this model. These data indicate that wound-induced FSCN1 upregulation and E-cadherin downregulation in the migrating wound front is impaired in diabetic wounds.

### GRHL3 directly regulates Fscn1 in wound-front keratinocytes and is required for global chromatin changes in response to wounding.

**Figure 7:**
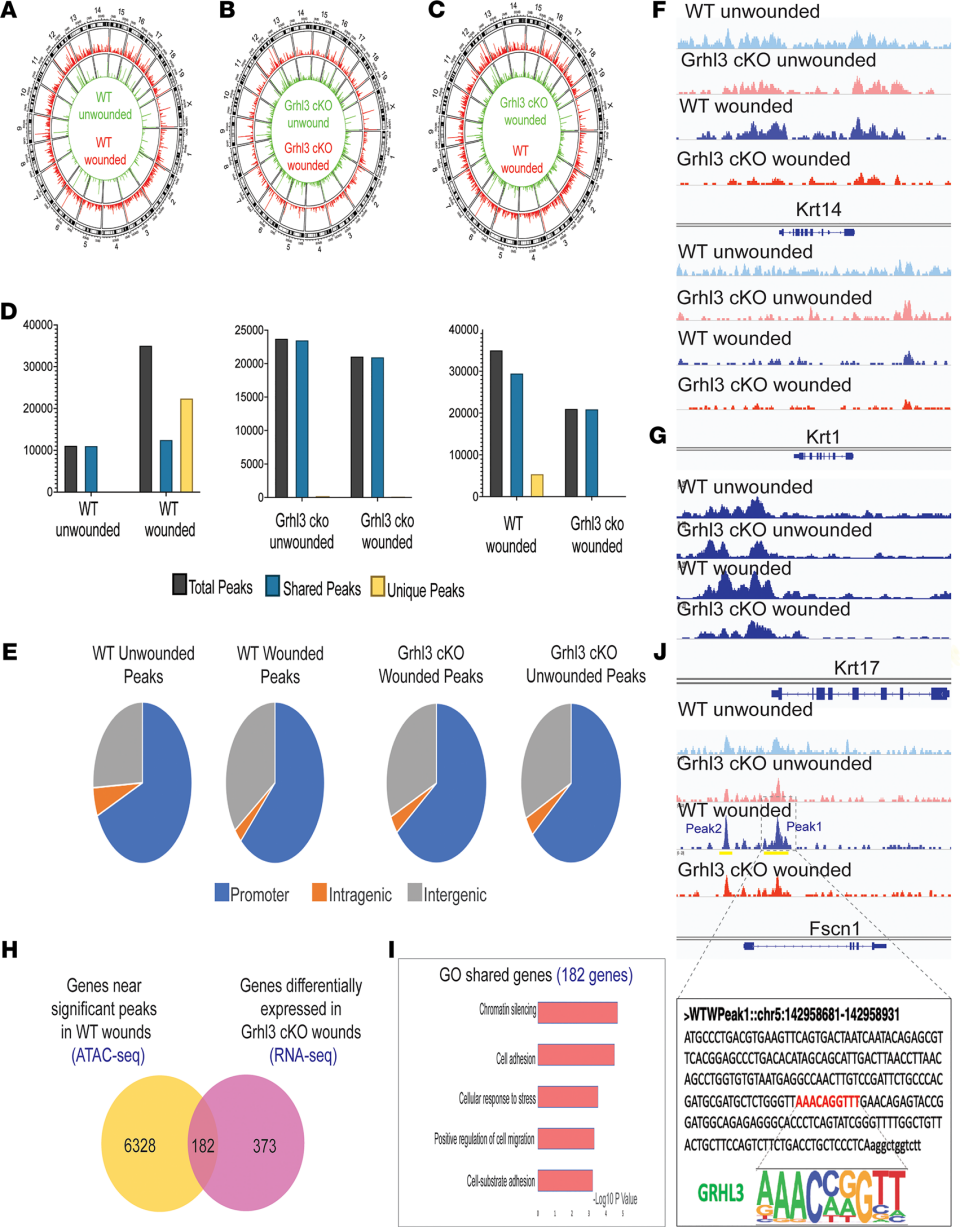
GRHL3 is required for global chromatin changes in response to wounding, and it directly regulates *Fscn1* in wound-front keratinocytes. (**A**–**C**) Landscape of chromatin accessibility from ATAC-seq analysis in unwounded and day-3 wounded keratinocytes in WT mice (**A**), unwounded and day-3 wounded keratinocytes in *Grhl3*-cKO mice (**B**), and day-3 wounded keratinocytes in *Grhl3*-cKO and WT mice (**C**). (**D**) Normalized number of total, shared, and unique peaks identified in unwounded and day 3 wounded keratinocytes in WT and *Grhl3*-cKO mice (*n* = 1 sample/condition; 4 wounds from 2 mice were pooled per condition per sample). (**E**) Genomic distribution of ATAC-seq peaks in unwounded and day 3 wounded keratinocytes in WT and *Grhl3*-cKO mice. (**F**) Sequencing tracks showing ATAC-seq peaks in keratin marker genes (*Krt14* and *Krt1*) in keratinocytes in unwounded and day 3 wounded keratinocytes in WT and *Grhl3*-cKO mice. (**G**) Sequencing tracks showing ATAC-seq peaks in a wound-response gene (*Krt17*) in keratinocytes in unwounded and day 3 wounded keratinocytes in WT and *Grhl3*-cKO mice. Dashed boxes and yellow lines indicate 2 markedly gained peaks in the *Krt17* gene in WT after wounding. (**H**) Venn diagram showing overlap between genes near significant ATAC-seq peaks (*P* < 0.05) in wounded keratinocytes in WT mice with genes that are differentially expressed by RNA-seq in wounded keratinocytes in *Grhl3*-cKO versus WT mice. (**I**) Gene ontology of overlapped genes (182) in **H**. (**J**) Sequencing tracks showing ATAC-seq peaks in the *Fscn1* gene in unwounded and wounded keratinocytes in WT and *Grhl3*-cKO mice. Boxed area shows one of the gained peaks (peak 1) on the *Fscn1* gene in WT mice after wounding; it also shows decrease in (peak 1) in *Grhl3*-cKO mice after wounding. Bottom panel shows a GRHL3 binding motif found in peak 1 in *Fscn1* gene.

We previously showed that GRHL3 binds differentially to chromatin in differentiating and migrating primary human keratinocytes (14, 15). Thus, we wished to study global chromatin accessibility in wound-front keratinocytes during reepithelialization and determine its dependence on GRHL3. We performed Assay for Transposase-Accessible Chromatin using sequencing (ATAC-seq) analysis on sorted keratinocytes isolated from day 3 wound margins and unwounded epidermis in WT and *Grhl3*-cKO as described above (Figure 4A). Global overview of enriched aligned reads, called peaks, in WT mice shows a robust increase in chromatin accessibility in keratinocytes of wounded compared with unwounded skin (Figure 7A). Surprisingly, in *Grhl3*-cKO mice, no major chromatin accessibility changes were observed in wounded keratinocytes compared with unwounded (Figure 7B). In fact, ATAC-seq peaks that were gained in response to wounding in WT mice generally failed to form in the absence of GRHL3 (Figure 7C), suggesting that GRHL3 is required for many changes in chromatin accessibility in response to wounding.

In WT mice, we observed 35,174 total peaks in wounded keratinocytes: 12,634 shared with unwounded and 22,540 peaks unique to the wound response (Figure 7D, left panel). By contrast, in *Grhl3*-cKO mice, we identified 21,156 total peaks in wounded keratinocytes, with only 116 peaks unique to wounding and the majority of the peaks shared with peaks found in unwounded keratinocytes (Figure 7D, middle panel). Although 21,068 peaks were shared in wounded keratinocytes between WT and *Grhl3*-cKO mice, 5553 peaks were exclusively gained in wounded keratinocytes in WT mice in response to wounding (Figure 7D, right panel). In addition, the genomic distribution of total peaks in wounded keratinocytes in WT mice shows a slight shift toward intergenic region compared with unwounded peaks; however, the majority of the peaks were still found in promoter regions after wounding (Figure 7E). Interestingly, the genomic distribution of peaks in wounded and unwounded keratinocytes in *Grhl3*-cKO mice is similar to the genomic distribution of peaks in wounded keratinocytes in WT mice (Figure 7E), suggesting that unwounded keratinocytes in *Grhl3*-cKO mice exhibit changes in chromatin accessibility prior to wounding. Together, these observations suggest that GRHL3 regulates global chromatin dynamics in the wound response.

Notably, the absence of *Grhl3* in keratinocytes did not significantly affect chromatin accessibility in epidermal genes such as the basal marker *Krt14* and the differentiation marker *Krt1* (Figure 7F). Consistent with our RNA-seq data (Figure 4), peaks that are significantly gained in the wound-response gene *Krt17* in wounded keratinocytes in WT mice were decreased in the absence of *Grhl3* (Figure 7G), supporting a broad impairment of the wound response in *Grhl3*-cKO mice (Figure 4, C and D). Next, we sought to determine the genes that are accessible in WT wounded keratinocytes and most affected by the loss of *Grhl3*. Thus, we overlapped genes that were found near the significant ATAC-seq peaks in WT wounded keratinocytes (6510 genes) with genes that were differentially expressed in *Grhl3*-cKO wounded keratinocytes when compared with WT wounded keratinocytes (555 genes), identifying 182 shared genes between these 2 data sets (Figure 7H). GO analysis of these 182 shared genes indicates enrichment of genes associated with chromatin silencing, cell adhesion, and cell migration — processes that are altered in response to wounding in the *Grhl3*-cKO mice (Figure 7I) — supporting the global wound-response effects of *Grhl3* loss in keratinocytes.

In the *Fscn1* gene, we noted that 2 peaks were significantly gained after wounding in WT mice; peak 2 is upstream of the promoter, and peak 1 is in the first intron (Figure 7J). In *Grhl3*-cKO mice, these peaks were unchanged after wounding (Figure 7J), consistent with decreased expression of *Fscn1* after wounding in the *Grhl3*-cKO mice. Broad peak analysis identified peak 1 as a significant peak in wounded keratinocytes compared with unwounded keratinocytes in WT mice. Motif analysis identified a strong GRHL3 motif in peak 1 (Figure 7J, bottom panel). Furthermore, reanalysis of our previous GRHL3 ChIP-seq experiments (14, 15) showed that GRHL3 binds to the promoter of the *Fscn1* gene in the mouse epidermis atE16.5 (Supplemental Figure 6B) and in migrating human keratinocytes in vitro (Supplemental Figure 6C). Collectively, these data indicate that GRHL3 binds to the *Fscn1* gene and regulates chromatin accessibility and *Fscn1* expression in wound-front keratinocytes during reepithelialization.

## Discussion

**Figure 8:**
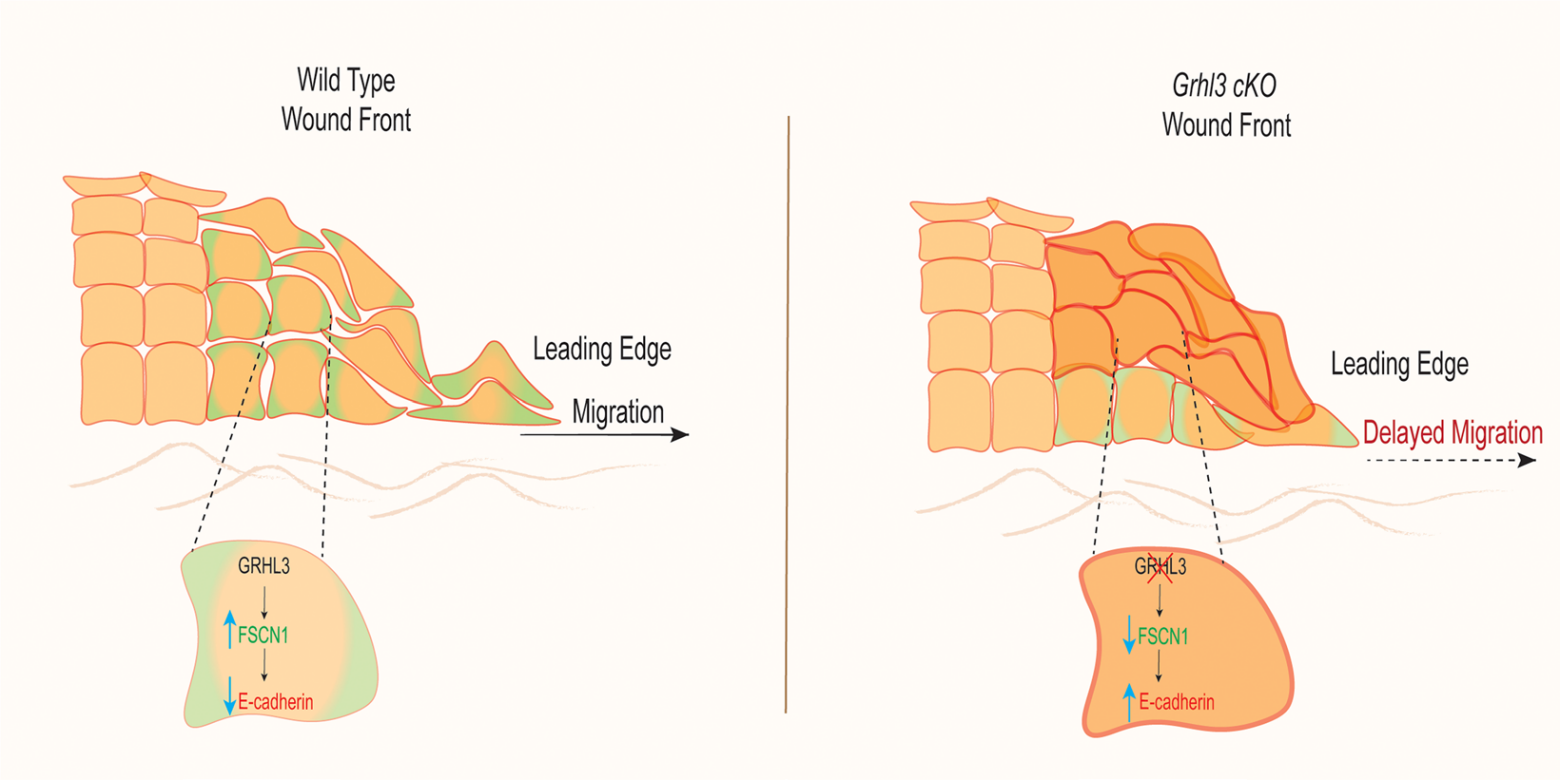
The GRHL3/FSCN1/E-cadherin pathway during wound-front keratinocyte migration. Schematic representation of the GRHL3/FSCN1/E-cadherin pathway in wound-induced keratinocyte migration. During acute wound reepithelialization, GRHL3 activates the expression of *Fscn1* in migrating follower cells, resulting in downregulation of E-cadherin and loosening of cell-cell adhesions, which is required for effective migration (left panel). In the absence of GRHL3, *Fscn1* is downregulated in follower cells, resulting in upregulation of E-cadherin and increased cell-cell adhesions, in turn slowing migration (right panel) and causing abnormal wound-front architecture.

The migrating epithelial wound front is an example of collective cell migration where a group of cells migrates as a cohesive epithelial sheet. Research in past years has brought understanding of the cellular and molecular mechanisms important for the function of leader cells, which are located at the edge of the migrating epithelial sheet and exert a pulling force (5). We know less about the regulation of distally located follower cells, which undergo distinct cellular modifications and require loosened cell-cell adhesion for effective migration of the wound front. Follower cells are regulated by Ephrin-B/EphB signaling, which drives both the loosening of adherens and tight junctions and the release of actomyosin tension during wound-induced epithelial migration (7). Here, we show that GRHL3, acting through a distinct mechanism, promotes loosening of adherens junctions by upregulating *Fscn1* in follower cells, which in turn decreases E-cadherin expression (Figure 8). The wound-front keratinocytes in adult *Grhl3*-cKO mice show decreased expression of *Fscn1*, increased expression of E-cadherin, increased cell-cell adhesion, abnormal architecture of the wound front, and delayed wound healing.

In embryonic wounds, GRHL3 promotes wound closure by directly upregulating RhoGEF19, a RhoA activator involved in planar cell polarity signaling in leader cells and a promoter of purse string formation (16). GRHL3 also promotes the activity of leader cells in embryonic eyelid closure (13). In barrier-disrupting adult epidermal injury, GRHL3 directly binds and suppresses immune-mediated inflammation genes and activates genes required for epidermal differentiation and barrier recovery (14), showing that GRHL3 is critical for the later stages of wound healing. We now show that GRHL3 is also important for the function of follower cells during the early reepithelialization stage in the healing of adult full-thickness wounds. Although our global gene expression and chromatin accessibility studies indicate that GRHL3 has a broad influence over the early wound response, we have focused here on GRHL3-regulated mechanisms in follower cells.

Previous studies have linked impaired wound reepithelialization to decreased cell proliferation and defective actin cytoskeleton organization in leader cells (17). Our data suggest the importance of a GRHL3/FSCN1/E-cadherin pathway for loosening of cell-cell adhesions in follower cells that promotes collective keratinocyte migration in wound closure (Figure 8). The key evidence are as follows.

First, the data show that GRHL3 activates the *Fscn1* gene in mouse and human keratinocytes in the wound front. The *Fscn1* mRNA (Figure 4I) and protein (Figure 5B) are downregulated in wound-front keratinocytes in *Grhl3*-cKO mice. Also, when GRHL3 is knocked down in migrating human keratinocytes in vitro, FSCN1 is downregulated (Figure 5D). Furthermore, analysis of our previously published GRHL3–ChIP-seq analyses from mouse embryonic epidermis (14) and migrating human keratinocytes (15) identified *Fscn1* as a direct GRHL3 target gene. Moreover, wound-induced chromatin accessibility in the *Fscn1* gene is impaired in *Grhl3*-cKO wound-front keratinocytes. *Fscn1* is also a direct target gene of the transcription factors SOX11 and SOX4 in embryonic epidermis and in wounded epidermis in adult mice (22); delayed wound reepithelialization in *Sox11* and *Sox4* gene-deleted mice is partially caused by *Fscn1* downregulation in the wounded epidermis (22).

Second, there is largely complementary expression of FSCN1 and E-cadherin in mouse (Figure 3F and Figure 5B) and human wound-front keratinocytes (Figure 5, C and D, and Figure 6C), consistent with FSCN1 downregulating E-cadherin in migrating keratinocytes. The complementary expression of FSCN1 and E-cadherin in migrating keratinocytes in vivo and in vitro is likely causally linked. Overexpression of FSCN1 in cancer cells leads to downregulation of E-cadherin and upregulation of EMT-promoting genes (25, 28, 29). In epithelial ovarian cancer cells, FSCN1 directly interacts with and increases the levels of SNAIL1, a repressor of E-cadherin expression (25). Here, we also found that knocking down *FSCN1* in migrating human keratinocytes resulted in increased E-cadherin expression at the cell membrane (Figure 5E). These studies support a causative link between FSCN1 and E-cadherin expression during epithelial cell migration.

Third, consistent with our model, we observe increased E-cadherin levels and cell-cell adhesion in wound-front keratinocytes in *Grhl3*-cKO mice (Figure 3). Furthermore, forced expression of *FSCN1* in *GRHL3*-knockdown cells mitigated the upregulation of E-cadherin (Figure 5F). Although we cannot rule out other mechanisms that could link GRHL3 to E-cadherin regulation, our data argue that GRHL3 acts, at least in part, via FSCN1 to regulate E-cadherin expression in migrating follower cells (Figure 8).

Although the GRHL3/FSCN1/E-cadherin pathway needs to be further investigated in chronic wounds, our work already suggests that it may be disrupted in conditions associated with impaired wound healing. We show an altered GRHL3 expression pattern in human chronic wounds (Figure 1). We also show that the FSCN1/E-cadherin pathway is altered in diabetic wounds, although this appears to be independent of GRHL3 (Figure 6). Therefore, abnormalities in the GRHL3/FSCN1/E-cadherin pathway could play a role in nonhealing wounds.

Work in organisms as divergent as worms, flies, and mammals suggests that Grainyhead transcription factors evolved to build and repair epithelial barriers (8, 9, 12, 30–34). Concomitant with the evolution of a complex cascade required for the repair of mammalian skin, Grainyhead factors, and GRHL3 in particular, seem to have taken on roles in multiple distinct mechanisms at different stages of wound healing. Here, we show that GRHL3 is required for collective cell migration at the early stages of adult wound healing. Previous data, mainly from normal embryonic processes and embryonic wound healing (13, 16, 30), have indicated a role for GRHL3 in leader cell mechanisms such as actin polymerization and filopodia projections. The current research shows that GRHL3 also plays a role in follower cells of the migrating wound front. Our previous work has shown a role for GRHL3 in the latest stages of wound healing, activation of terminal differentiation genes, and the repair of the barrier and suppression of inflammation (14). The ability of GRHL3 to target different genes depending on cellular context relates in part on the underlying enhancer landscape, which changes between proliferating, differentiating, and migrating keratinocyte, as shown for human keratinocytes (15). In addition, GRHL3 collaborates with different chromatin modifiers to control gene expression and chromatin conformation (35). These include the histone methyltransferase Trithorax (36) and the histone deacetylase REST (15), which mediate, respectively, GRHL3-regulated activation and repression.

## Methods

### Mice.

Mice were housed and maintained in accordance with protocols approved by the UCI’s IACUC. Transgenic mouse strains used in this study were on the C57BL/6J background: *Grhl3^ﬂ/ﬂ^*, *Krt14Cre*;*Grhl3^ﬂ/ﬂ^*, and *Grhl3*-Cre;LacZ (14). Skin-specific deletion of *Grhl3* in C57BL/6J mice was generated by crossing *Grhl3* ﬂoxed mice with Tg (KRT14-cre)1Amc/J (stock no. 004782). *Grhl3-Cre*;*LacZ* reporter mice were purchased from MMRRC. C57BL/6J-SCID mice were purchased from The Jackson Laboratory (stock no. 001913).

### Mouse excisional wounds.

Age-matched (8 weeks) *Krt14-Cre*-*WT* and *Krt14-Cre-Grhl3^fl/fl^* littermates (males and females) were used for the study. Prior to wounding, mice were anesthetized with ketamine/xylazine according to UCI’s IACUC guidelines. Dorsal back skin was shaved, and hair was removed using Nair cream. Small full-thickness wounds were created using (4 mm) biopsy punch on the shaved back skin. Wounded mice were placed on a heating pad and monitored until full recovery. For wound-closure rate, mice were anesthetized at the indicated time points, and pictures of the wounds were obtained. For BrdU-labeling experiments, mice were injected with 0.5 mg/mL of BrdU and sacrificed 3 hours after the injection. BrdU^+^ cells in the hyperproliferative epidermis and the migrating wound front were counted.

### Wound collection, histology, and IHC.

Wounded mice were euthanized, and wounded skin was collected 1, 3, 5, 7, and 10 days after wounding. For histology, wounds were dissected and fixed with 10% formalin for 48 hours at 4°C, followed by ethanol dehydration. Paraffin-embedded wounds were sectioned (8 μm thick) with a microtome. H&E staining was performed as previously described (34). For IHC, paraffin-embedded wound sections were stained following antigen-retrieval as previously described (14). Primary antibodies used were anti-βGal (ab9361, Abcam), anti-BrdU (ab6326, Abcam), and anti-GRHL3 (HPA059960-100UL, MilliporeSigma).

For immunofluorescence, fresh-frozen wound samples were embedded in OCT compound and sectioned (8 μm thick) with a cryostat. Slides were fixed in cold acetone for 10–13 minutes at room temperature (RT). After 3 washes with PBS, slides were fixed with 4% PFA for 10 minutes. Slides were then permeabilized with permeabilization buffer (Triton X in PBS) for 10–15 minutes and blocked with blocking buffer (1× PBS+ 2%BSA) for 1 hour. For primary antibody, slides were incubated with anti–E-cadherin (24E10, Cell Signaling Technology), anti-FSCN1 (HPA005723, MilliporeSigma), anti-CLDN1 (ab15098, Abcam), anti-GRHL3 (HPA059960-100UL, MilliporeSigma), and anti-KRT17 (D12E5, Cell Signaling Technology) overnight at 4°C. After several washes with PBS, slides were incubated with secondary antibody (Alexa Fluor 488/ Alexa Fluor 594) for 1 hour at RT in the dark. Stained slides were mounted with a mounting DAPI solution for nuclear staining. Slides were imaged with a Keyence BZ-X710 all-in-one fluorescence microscope.

For Masson’s trichrome staining, paraffin-embedded wound sections (8 μm thick) were de-paraffinized, hydrated, and stained with Trichrome Stain Kit (HT15-1KT, MilliporeSigma). For Collagen Index measurements in wound skin tissues, ImageJ software was used (NIH).

### TEM and toluidine blue staining.

Wound tissues were fixed in 0.25% glutaraldehyde for 1–2 days. Samples were washed with cacodylate buffer, postfixed with 1% osmium tetroxide, dehydrated through several ethanol series, and embedded in resin. Sections (1 nm) were contrasted with uranyl acetate and lead citrate, and they were examined on FEI TECNAI SPIRIT transmission electron microscope. Thin (100 nm) resin embedded sections were stained with Toluidine blue as previously described (37).

### Human wound samples.

Punch biopsies of healing and nonhealing wounds were collected for clinical purposes under UCI standard medical care. Samples were deidentified prior to use. Paraffin embedded samples were sectioned (8 μm) for histological analysis.

### Human acute wound model.

Human grafts (collected from facial, chest, and abdominal skin) are discarded surgical tissues that were deidentified prior to collection and use. All dissections and collections were done in fresh, cold 1× PBS. S.c. fat and fascia were removed, and dermis was trimmed from lower dermal fat. Tissues were rinsed again in 1× PBS and grafted onto a 1 cm^2^ excision in the back skin of immunodeficient mice (C57BL/6J-SCID). Xenografted skin were left to heal for 30 days prior to wounding. Full-thickness wounds were created using biopsy punch (2 mm). Wound tissue was collected 3 days after wounding and embedded in OCT for immunofluorescence analysis. 

### Diabetic mouse wound model.

Adult C57BL/6J mice (males and females) were fed high-fat diet (HFD) for 3 months. Fasting blood glucose was measured using a blood glucose monitor (OneTouch-Verio glucose meter) before wounding. Diabetic mice (fasting blood glucose > 200 mg/dL) were anesthetized with ketamine/xyline according to according to UCI’s IACUC guidelines. Small full-thickness wounds were created using (4 mm) biopsy punch on the shaved back skin as described above. Wound tissue was collected 3 days after wounding and embedded in OCT for immunofluorescence analysis.

### Protein extraction and Western blotting.

Wound margins (1 mm) were collected and snap-frozen in liquid nitrogen. Following tissue grinding, samples were lysed in RIPA lysis buffer (Bio-Rad) and spun for 15 minutes at 4°C. Supernatant was collected and purified using MilliporeSigma purifying columns. Protein concentration was determined using a Bradford assay (Bio-Rad). Equal amount of protein samples (50 μg/mL) were separated by SDS-page and transferred to nitrocellulose membranes. Blots were blocked with 5% nonfat milk in TBST for 1 hour at RT. Following blocking, blots were incubated with anti–E-cadherin (24E10, Cell Signaling Technology) and anti-GAPDH (D16H11, Cell Signaling Technology) antibodies at 4°C overnight. After 3 washes with TBST, blots were incubated with secondary antibody (HRP, 1:1000) for 1 hour at RT. Bands were developed by adding 1 mL of Westfemto Illuminata (Bio-Rad) and imaged using Genesys. Band intensities were measured using ImageJ software. E-cadherin band measurements were normalized to GAPDH.

### Cell transfection and in vitro scratch assay.

HaCaT cells were cultured in 24-well plates and 8 mm glass chambers in Keratinocytes-SFM media (Thermo Fisher Scientific). Lipofectamine RNAi Max (Invitrogen) in OptiMEM medium was used for transfections. Confluent cells were transfected with 30 nM of siRNA: scrambled siRNA (siRNA_ID: D-001810-10-05, Dharmacon), Grhl3 siRNA (siRNA_ID: L-014017-02, Dharmacon), *FSCN1* siRNA (siRNA_ID: s13207, Invitrogen), and *pRP-EGFP-hFSCN1* plasmid (500 ng of total DNA; Vector_ID:vb201205-1001gxv, Vector Builder). Transient transfection using lipofectamine 2000 was performed as previously described (15). Transfection media was replaced after 15–20 hours, and a scratch was made with a pipet tip in the center of the well. Cells were fixed after 12–15 hours with cold methanol and 4% PFA for immunofluorescence analysis. Two independent experiments were conducted, with 3–4 technical replicates per condition.

### qPCR.

Epidermal cells were isolated from the wound margin 3 days after wounding from WT and *Grhl3*-cKO mice. RNA was extracted using Direct-zol RNA Miniprep Kits (Zymogen). cDNA synthesis was prepared using iScript cDNA Synthesis Kit (Bio-Rad). qPCR reaction was prepared using SsoFast for Probes and SsoFast EvaGreen (Bio-Rad). Primers sequences of *Grhl3* (Forward, 5′-CCCCTGAGCAGT TGGAAT A-3′; Reverse, 5′-TGG CCACACTGACAAGAG-3′), *Cdh1* (Forward, 5′-GGTCATCAGTGTGCTCACCTCT-3′; Reverse, 5′-GCTGTTGTGCTCAAGCCTTCAC-3′), *ArhGef19* (Forward, 5′-GAGGTCAAGAGCACCAGTGAGA-3′; Reverse, 5′-GTTGGTGACGTAAGGCAGGTAG-3′), and mouse GAPDH (purchased from Applied Biosystems). RT-PCR was performed using master mixes in CFX384 Real-Time PCR.

### Flow cytometry and FACS.

Wound margins (1 mm margins) and distal unwounded skin were collected, s.c. fat was removed with a scalpel, and tissues were placed epidermis facing up on 0.25% trypsin-EDTA (Thermo Fisher Scientific) at 37°C for 2 hours on a shaker. Scraped epidermis was diced in 2 mL of media (Epilife). Cell suspension was filtered through 70 μm followed by 40 μm cell strainers. Single cells were washed with Red-cell lysis buffer followed by PBS, before being blocked with FACS buffer (1%–2% BSA in PBS) for 5 minutes on ice. Fluorophore-conjugated antibodies: CD49F-PE (Bioscience, 12-0495-82), CD45-APC (Bioscience, 17-0451-82), CD31-APC (Bioscience, 17-0311-82), and TER119-APC (Bioscience, 48-5921-82) were added and incubated in the dark for 30 minutes on ice. Isotype controls of the indicated fluorophore were used as staining controls. Stained cells were suspended in 400 μL of DAPI/FACS buffer and filtered through 40 μm strainer before sorting. Samples were processed using BD LSR II flow cytometer and sorted into 350 μL RLT-BME lysis buffer (Qiagen). RNA extraction was performed using Microelute RNA kit (Qiagen). RNA quality was assessed using Agilent Bioanalyzer, and concentration was measured by PicoChip. Samples with high RIN score >8 were used for sequencing.

### RNA-sequencing and analysis.

Sequencing libraries (Single-Read-100) were prepared at UCI’s GHTF facility using Illumina ClonTech mRNA sample preparation kit. Libraries were sequenced on Illumina HiSeq 4000 (50 million reads per sample). Sequencing quality of sequencing data (FastQ files) was determined using FASTQC. Reads were aligned to the mouse genome (mm10 build) using Kallisto version 0.46.2. A list of differentially expressed genes was generated using EdgeR version 3.24.3 in R version 3.5.2. GO was generated using DAVID. HOMER and MEME were used to identify de novo motifs and enriched GRHL3 motifs in candidate genes, respectively.

### ATAC-seq.

Keratinocytes (100,000 cells/sample) from 1 mm wound margins or unwounded skin were sorted into 300 μL of FACS buffer. Cells were spun at 500*g* for 5 minutes at 4°C and were then washed with cold 1× PBS. In total, 50 μL of cold lysis buffer (10 mM Tris hydrochloride, 3 mM MgCl_2_, 10 mM NaCl, 0.1%MgCl_2_, 10% NP-40; MilliporeSigma) was added to pelleted cells and pipetted up and down to resuspend cells, followed by centrifugation at 500*g* for 10 minutes at 4°C. The supernatant was discarded and 50 μL of transposition mix (2× Tagment DNA Buffer, Tn5 Transposase in nuclease-free H_2_O) was added to the cell pellet and incubated for 30 minutes at 37°C. Following transposition, DNA was isolated using (Qiagen MinElute Reaction Cleanup kit). DNA was eluted in 10 μL of Elution Buffer (catalog 28206). DNA quality was determined using High-Sensitivity DNA assay and PCR amplification. Library generation (Paired-end sequencing of 100 bp DNA fragments) was performed using (Illumina sample preparation kit) and then sequenced on Illumina HiSeq 4000 (>70 million reads per sample). Quality profiling was performed on sequencing files using FASTQC version 0.11.9. Adaptors were removed and sequencing were trimmed using Trimmomatic version 0.39 (a flexible trimmer for Illumina Sequence Data. Bioinformatics, btu170.). Properly paired reads were then aligned with mouse mm10 genome using bowtie2 version 2.2.5 with the “very sensitive” parameter (38). MACS2 version 2.1.4 was used for peak calling and differential peak calling under standard conditions with a sequencing tag size of 100. Bigwig files were generated with UCSC-bedgraphtobigwig version 366 and were visualized using IGV version 2.6.2. Custom scripts were built in R version 3.5.2 to create circos plots using the R package circlize. Samtools version 1.9 and Bedtools version 2.29.2 were used for various processing and to compare peaks between samples to find unique and shared peaks (39). MEME CentriMo was used under standard conditions for motif identification using an established GRHL3 motif.

### Data and software availability.

All data sets have been deposited in the National Center for Biotechnology Information/Gene Expression Omnibus (GEO) under accession GSE172161.

### Statistics.

One-way ANOVA and unpaired 2-tailed Students’ *t* tests were performed in GraphPad Prism 6.0 software. Data are expressed as mean ± SEM, and *P* values were reported such that *P < 0.05 and **P < 0.005.

### Study approval.

This work was conducted with approval of the UCI Human Research Protections Program. Mice were housed and maintained in accordance with protocols approved by the UCI’s IACUC. All mouse experiments in this study were preformed under UCI’s IACUC guidelines and approval.

## Author contributions

GK and BA conceived the overall project. GK, SR, BP, and AB performed and analyzed experiments. SV and RHK performed bioinformatic analysis. APT and PHW generated and analyzed diabetic mouse models. RR and MVP generated and analyzed mice with human xenografts. PHW, MVP, BA, and GK analyzed data. GK wrote the manuscript with editorial input from all other authors.

## Supplementary Material

Supplemental data

Supplemental table 1

## Version 1 09/08/2021

Electronic publication
