# Supplementary material for: GRHL3 activates FSCN1 to relax cell-cell adhesions between migrating keratinocytes during wound reepithelialization
Source: JCI Insight. 2021 Sep 8;6(17):e142577. doi: 10.1172/jci.insight.142577 (PMC8492311; doi:10.1172/jci.insight.142577)
Supplement: Supplemental data [file jciinsight-6-142577-s228.pdf]

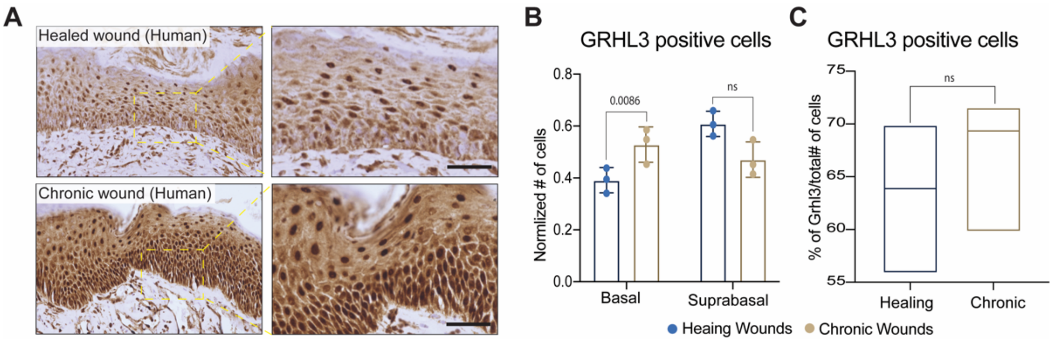

Figure S1. GRHL3 is upregulated in wound-front keratinocytes of acute wounds; its expression is altered in human chronic wounds

(A) Immunohistochemistry of GRHL3 expression (anti-GRHL3) in tissue sections from healing wounds and chronic wounds (additional patients to the patients shown in Fig. 1) (left panels). Higher magnifications of the wounded epidermis (right panels) (Scale bar = 36 um). (B) Ratio of basal and suprabsal keratinocytes that express GRHL3 in healed and chronic wounds (N = 3/group). Data are Mean ± SEM. (C) GRHL3-positive cells in healed and chronic wound epidermis (N = 3/group). Data are Mean ± SEM. Statistical significance was determined using student T-test (***p value* = 0.0086).

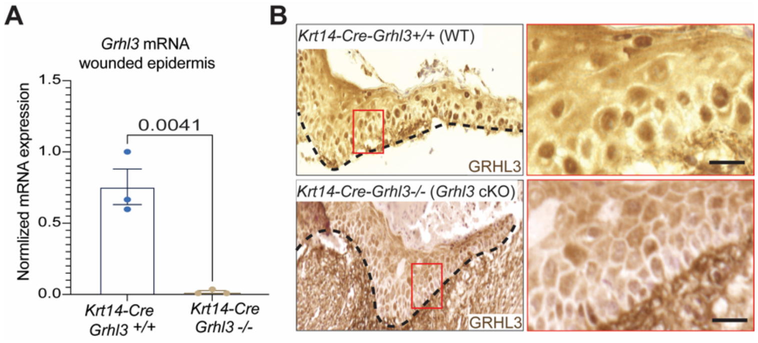

Figure S2. GRHL3 is required for proper architecture and function of the collectively migrating wound front

(A) QPCR analysis showing normalized expression of *Grhl3* mRNA in wounded epidermis in WT and *Grhl3 cKO* mice (N = 3/genotype). Data are Mean ± SEM. Statistical significance was determined using student T-test (***p value* = 0.0041). (B) Immunohistochemistry of GRHL3 expression (anti-GRHL3) in wound section from WT and *Grhl3 cKO* mice 3 days after wounding (left panels). Dashed lines indicate the epidermis-dermis junction. Higher magnification of the wound leading edge (red box) in WT and *Grhl3 cKO* mice (right panels). Scale bar 30 um.

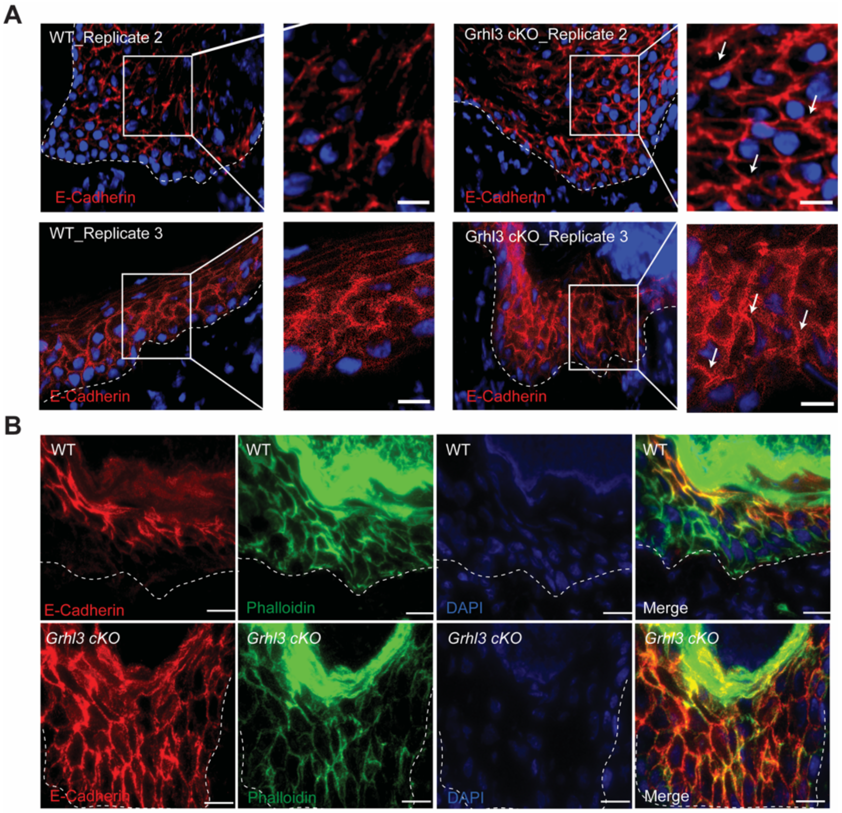

Figure S3. GRHL3 mediates decreased E-cadherin expression and loosening of cell-cell adhesion in the migrating wound front

(A) Immunofluorescence analysis of E-cadherin (anti-E-cadherin) in day 3 wound sections collected from WT and *Grhl3* cKO mice (N = 2/genotype, additional replicates). Dotted line indicates the wound front epithelium. White box indicates the magnified area on the right. White arrows point to the higher E-cadherin expression at the cell membrane in *Grhl3 ckO* mice (scale bar = 30 um). (B) Immunofluorescence analysis of E-cadherin (anti-E-cadherin), F-actin (phalloidin), and DAPI in day 3 wound sections collected from WT and *Grhl3* cKO mice (Scale Bar 25 um). The co-staining of Phalloidin and E-cadherin indicates there is increased cell surface expression of E-cadherin in the wound front of *Grhl3* cKO mice.

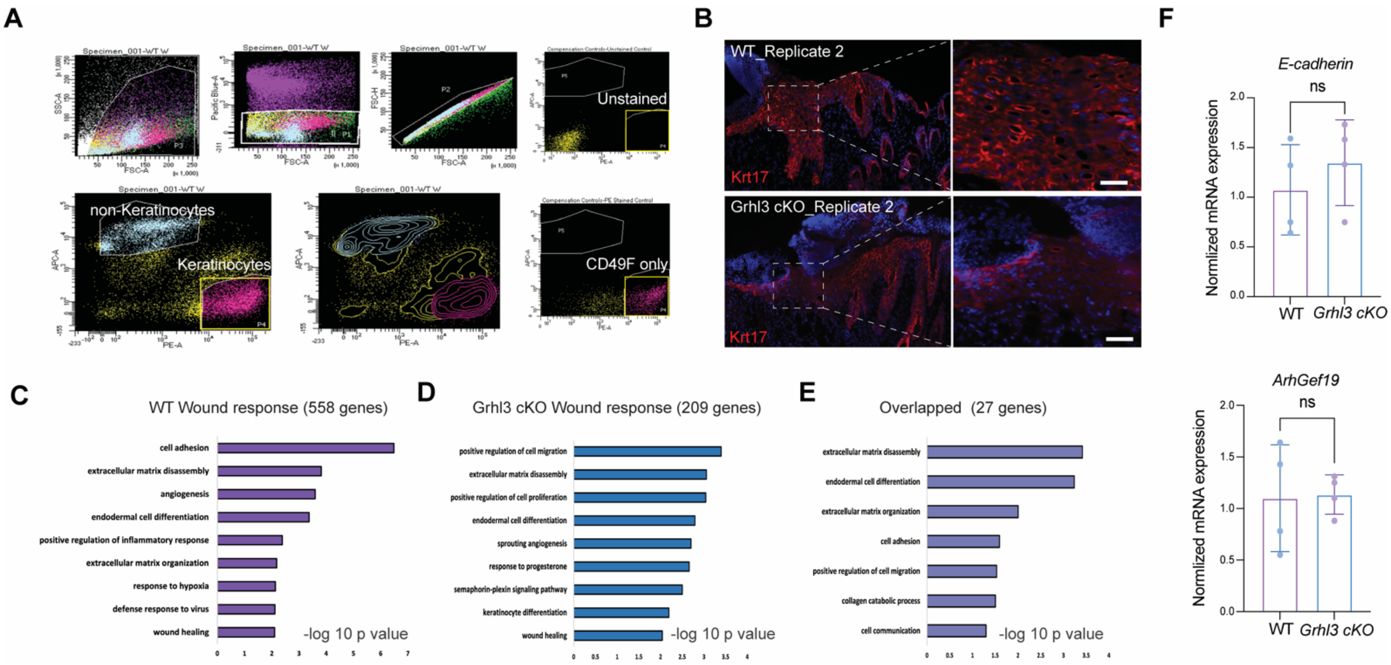

Figure S4. GRHL3 regulates the expression of factors that modulate E-cadherin levels and cell-cell adhesion in the wound front

(A) Fluorescence-activated cell sorting (FACS) gating strategy to isolate wound-front keratinocyte from day 3 wounded epidermis. (B) Immunofluorescence analysis of Keratin-17 (anti-Krt17) in wound sections from WT and *Grhl3* cKO mice (additional replicate). White boxes indicate the magnified area in the side panels (scale bar = 45 um). (C-E) Gene Ontology of differentially expressed genes in (C) WT wounded vs. WT unwounded (D) *Grhl3* cKO wounded vs. *Grhl3* cKO unwounded, and (E) genes that overlap between C and D. (F) QPCR analysis showing normalized *Cdh1* (left panel) mRNA and ArhGef19 (right panel) mRNA expression levels in wounded epidermis in WT and *Grhl3 cKO* mice (N = 4/genotype). Data are Mean ± SEM.

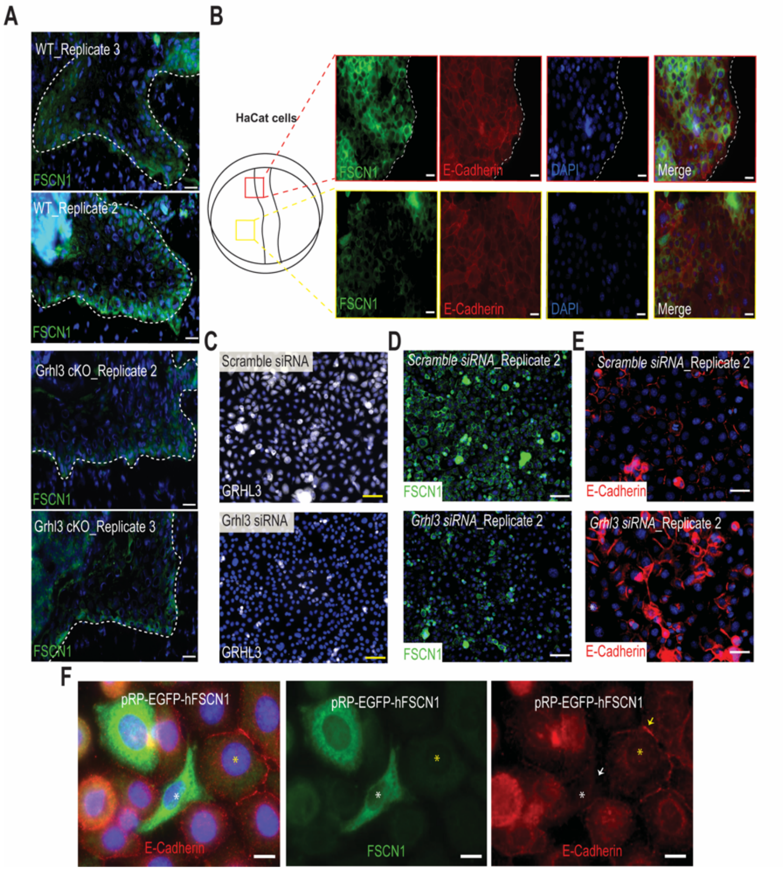

Figure S5. GRHL3 upregulates FSCN1 in migrating wound-front keratinocytes

(A) Immunofluorescence analysis of FSCN1 (anti-FSCN1) in day 3 wound sections in WT and *Grhl3* cKO mice (additional replicates). Dotted lines indicate the basis of the wound front (scale bar = 40 um). (B) Schematic of the *in vitro* scratch wound assay, with black boxes indicating the location of non-migrating and migrating HaCaT cells (top panel). Immunofluorescence analysis of FSCN1 (Green), E-cadherin (Red), and DAPI (Blue) in non-migrating and migrating HaCaT cells (lower panels). Dashed lines indicate migrating leading edge (scale bar = 70 um). (C) Immunofluorescence analysis of GRHL3 (Gray) and DAPI (Blue) after transient transfection with scrambled siRNAs (top) and *Grhl3* siRNAs (bottom) (scale bar = 200 um). (D) Immunofluorescence analysis of FSCN1 (Green) and DAPI (blue) in HaCat cells after transient transfection with scrambled siRNAs (left) and *Grhl3* siRNAs (right) (scale bar = 200 um). (E) Immunofluorescence analysis of E-cadherin (Red) and DAPI (blue) in HaCat cells after transient transfection with scrambled siRNAs (left) and *Grhl3* siRNAs (right) (scale bar = 70 um). (F) Immunofluorescence analysis of FSCN1 (Green), E-cadherin (Red), and DAPI (Blue) in migrating HaCaT cells transfected with h*Fscn1* plasmid. White asterisks indicate cells with FSCN1 overexpression. Yellow asterisk indicates a cell with low FSCN1 expression. White arrows indicate the absent E-cadherin protein at the cell membrane. Yellow arrow indicates the expression of E-cadherin at the cell membrane (Scale Bar = 10 um).

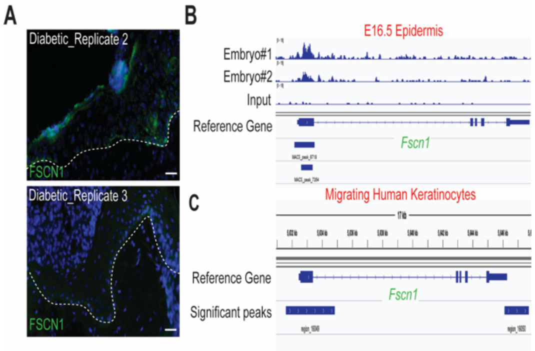

Figure S6. GRHL3 is required for global chromatin changes in response to wounding and it directly regulates *Fscn1* in wound-front keratinocytes

(A) Immunofluorescence analysis of FSCN1 (anti-FSCN1) in two additional diabetic mice at day 3 after wounding (scale bar = 200 um). Dashed lines indicate the basis of the migrating epithelium in the wound front. (B) Sequencing tracks showing significant GRHL3 ChIP-seq peaks on the *Fscn1* gene in WT mouse embryonic epidermis at E16.5. (C) Sequencing tracks showing significant GRHL3 ChIP-seq peaks on the *FSCN1* gene in migrating primary human keratinocytes in *vitro*. Blue bars in lower tracks indicate significant peaks.
